# Revisiting implementation of multiple natural enemies in pest management

**DOI:** 10.1038/s41598-022-18120-z

**Published:** 2022-09-02

**Authors:** Weam Alharbi, Simran K. Sandhu, Mounirah Areshi, Abeer Alotaibi, Mohammed Alfaidi, Ghada Al-Qadhi, Andrew Yu Morozov

**Affiliations:** 1 Department of Mathematics, Faculty of science, University of Tabuk, Tabuk, 71491 Saudi Arabia; 2 School of Computing and Mathematical Sciences, University of Leicester, Leicester, LE1 7RH UK; 3 Department of Biology, University College of Duba, University of Tabuk, Tabuk, 71491 Saudi Arabia; 4 Laboratory of Behaviour of Lower Vertebrates, Institute of Ecology and Evolution, Moscow, 119071 Russia

**Keywords:** Computational models, Ecology, Ecological epidemiology, Ecological modelling, Population dynamics, Evolution, Coevolution, Ecological modelling, Applied mathematics

## Abstract

A major goal of biological control is the reduction and/or eradication of pests using various natural enemies, in particular, via deliberate infection of the target species by parasites. To enhance the biological control, a promising strategy seems to implement a multi-enemy assemblage rather than a single control agent. Although a large body of theoretical studies exists on co-infections in epidemiology and ecology, there is still a big gap in modelling outcomes of multi-enemy biological control. Here we theoretically investigate how the efficiency of biological control of a pest depends on the number of natural enemies used. We implement a combination of eco-epidemiological modelling and the Adaptive Dynamics game theory framework. We found that a progressive addition of parasite species increases the evolutionarily stable virulence of each parasite, and thus enhances the mortality of the target pest. However, using multiple enemies may have only a marginal effect on the success of biological control, or can even be counter-productive when the number of enemies is excessive. We found the possibility of evolutionary suicide, where one or several parasite species go extinct over the course of evolution. Finally, we demonstrate an interesting scenario of coexistence of multiple parasites at the edge of extinction.

## Introduction

Biological control is currently considered an efficient tool to reduce and/or eradicate a large variety of pest species across the world. This is an environmentally friendly and less costly alternative to conventional chemical control using pesticides, insecticides, or fungicides^1–5^. In many cases, biological control agents are parasites, parasitoids, or pathogens: by infecting their host, they reduce fitness and increase the mortality rate of the target pest species^6–8^. Usually, a biological control agent is a specialist (e.g. a host-specific parasite) since the implementation of a generalist might affect other non-target species in ways which are hard to predict, potentially damaging the ecosystem^9,10^. However, utilising a specialist natural enemy has the fundamental drawback that it can hardly eradicate the target pest unless the former is highly vulnerable to an Allee effect^11^. Moreover, the specialist control agent goes extinct once its target pest resource is eliminated, and a further resurgence of the pest due to its occasional re-introduction from neighbour sites is possible^9,12^. The other difficulty is the eventual evolution of life-history traits of the control agent, making it less deadly for the target species. For example, pathogens used as biocontrol tools may evolve in a way that their virulence reaches some intermediate evolutionary stable values to enable the optimal exploitation of the host^13–15^. A notable example is the well-documented co-evolution of the myxoma virus used to control populations of the European rabbit in Australia. The initial mortality of 99.8% of the host has largely dropped, providing a stable co-existence between the pathogen and the host^16^ at still high population numbers of the European rabbit.

To partly compensate for the pre-mentioned negative effects of the use of a specialist natural enemy, a promising approach is implementing a multi-enemy assemblage rather than a single biocontrol agent^17–21^. An important practical example of implementing multiple enemies is the biological control of the red palm weevil, *Rhynchophorus ferrugineus*, which is a pest insect that infests date palms and eventually kills them. Red palm weevil significantly damages date production in the Middle East and around the world^22,23^. It was shown that a joint application of fungus and nematode treatments of red palm weevil resulted in higher mortality as well as a lower fitness of the target pest^2^. However, in the mentioned example of the biological control of the red palm weevil, as in several similar study cases (e.g. the biological control of black vine weevil^24^), a short-term, i.e. based on a single generation, laboratory experiments demonstrating increased mortality can be misleading since they do not take into account long-term evolution or environmental feedback related to host-parasite interaction^18,25^. Mathematical modelling is considered to be an efficient tool for elucidating the effects of long-term co-evolution of host-parasite systems with complex environmental feedback.

Mathematical modelling of co-infections has been intensively elaborated in recent years and is a continually growing area of research^15,26–28^. An important generic finding from theoretical models is that the overall virulence (i.e. the increase in mortality due to parasites) in the case of multiple infections usually increases as compared to the single infection scenario due to a competitive advantage of more virulent parasites^25,26^. This prediction is encouraging for using the multi-enemy assemblage paradigm in pest management. However, the central practical question, which remains unclear, is about the alteration of the population size of the target pest following bio-control, i.e. whether the usage of multiple agents will greatly reduce the negative impact of the pest on the environment. In fact, the existing theoretical studies of co-infections are mostly focused on the evolution of virulence, disease prevalence or the derivation of the basic reproduction number^25,26,28–31^. For example, in some co-infection models, the population size of a host is kept constant for simplicity, thus making it impossible to conclude if we should expect a reduction of the pest abundance^25^. Another important aspect not properly addressed in the literature is: would an increase in the number of biocontrol agents used (i.e. the biodiversity of parasites) translate itself into a more efficient biocontrol of the target species, i.e. whether ‘the more, the better’ principle holds^32^?

This study is conceived to partly cover the pre-mentioned gaps. We theoretically investigate the efficiency of pest management under the multiple natural enemies framework in the case where all control agents are different species. We explore the outcomes of biocontrol on both short-term (population) and long-term (evolutionary) time scales. To model the evolutionary time scale, we apply the Adaptive Dynamics framework^13^, with the fast epidemiological time scale using the classical SI modelling framework^31^. Adaptive dynamics^33,34^ (based on the so-called canonical equation) is a combination of game theory and population dynamics, evolutionary outcomes emerge following a large number of consecutive small-sized and rare mutations, their further invasion and replacement of the resident population^35–37^. Unlike previous studies, we allow for an arbitrary number of types of parasites (pathogens), which can co-infect a target (pest) species. We found that progressively adding new parasite types in the system may only have a limited effect on reducing pest numbers, even though the overall virulence increases. We show that a reduction of the transmission rate a parasite due to the presence of other parasite types can impede the efficiency of biocontrol. We found the possibility of an evolutionary suicide in the system where one or more co-infecting pathogens would eventually go extinct over the course of evolution. We show the scenario where all co-infecting pathogens can coexist at the edge of extinction by showing high-amplitude stochastic oscillations of population density. Finally, we briefly discuss the practical consequences of our study for the multi-enemy biological control by parasites, in particular the biological control of the red palm weevil.

## Methods

### Model equations

Our host-parasite mathematical model involves the following host population components: ‘susceptible’ hosts denoted by (*S*), and hosts infected by *k* distinct types of parasites ($k=1,2,...,n$), the corresponding population numbers of infected hosts are denoted by $I_{i_1,i_2,...,i_k}$, where each index $i_j$ can take a value from 1, ..., *n* (to avoid repeated counting of the same infection configuration, we require throughout the paper that $i_1<i_2<i_3<...<i_k$). Altogether our model contains $2^n$ equations for the densities of *S* and $I_{i_1,i_2,...,i_k}$. Importantly, we consider that parasites are distinct species, or are very different strains of the same species, which can not mutate into each other, so we do not explore the effects of kin selection. Note that our model is an extension of previous host-parasite models with multiple infections, such as the model by Choisy and de Roode^25,38^, or, more generally, well-known co-infection models in epidemiology^31,39^. The flowcharts of the model for $n=2$ are shown in the supplementary material (SM2).

The model equations for *S* and $I_{i_1,i_2,...,i_k}$ (with $k=1,...,n$) read as follows:

1 $$\begin{aligned}{}&\begin{aligned} \frac{dS}{dt}=F(N)S-S\left( \sum _{j=1}^{n}\phi _{i_j}+\mu \right) , \end{aligned} \end{aligned}$$

2 $$\begin{aligned}{}&\begin{aligned} \frac{dI_{i_k}}{dt}=S\phi _{i_k}-I_{i_k}\left( \sum _{j=1,i_j\ne i_k}^{n}\phi _{i_j}+\mu +\alpha _{i_k}\right) , \end{aligned} \end{aligned}$$

3 $$\begin{aligned}{}&\begin{aligned} \frac{dI_{i_{k_1},i_{k_2},...,i_{k_{{\bar{n}}}}}}{dt}=\sum _{i_j \in \{i_{k_1},i_{k_2},...,i_{k_{{\bar{n}}}}\}} I_{\{i_{k_1},i_{k_2},...,i_{k_{{\bar{n}}}}\}\backslash {i_j}}\phi _{i_j}-I_{i_{k_1},i_{k_2},...,i_{k_{{\bar{n}}}}}\left( \sum _{i_j \notin \{i_{k_1},i_{k_2},...,i_{k_{{\bar{n}}}}\}}\phi _{i_j}+\mu +\alpha _{{i_{k_1},i_{k_2},...,i_{k_{{\bar{n}}}}}}\right) , \end{aligned} \end{aligned}$$

4 $$\begin{aligned}{}&\begin{aligned} \frac{dI_{1,2,...,n}}{dt}=\sum _{j \in \{1,2,...,n\}} I_{\{1,2,...,n\}\backslash {j}}\phi _{j}-\left( \mu +\alpha _{{1,2,...,n}}\right) I_{1,2,...,n}{,} \end{aligned} \end{aligned}$$

where

5 $$\begin{aligned} \begin{aligned}{}&\phi _{i_j}=I_{i_j}\beta ^{{i_j}}_{i_j}+\sum _{j_1=1,j_1\ne j}^{n}\Biggl (\beta ^{{i_j}}_{i_j,i_{j_1}}I_{i_j,i_{j_1}}\\&\quad +\sum _{j_2=j_1+1,j_2\ne j}^{n}\left( \beta ^{{i_j}}_{i_j,i_{j_1},i_{j_2}}I_{i_j,i_{j_1},i_{j_2}}+...+\sum _{j_{n-1}=j_{n-2}+1,j_{n-1}\ne j}^{n}\beta ^{{i_j}}_{{i_j,i_{j_1},i_{j_2},...,i_{j_{n-1}}}}I_{i_j,i_{j_1},i_{j_2},...,i_{j_{n-1}}}\right) \Biggr ), \end{aligned} \end{aligned}$$

is the infection rate of strain $i_j$ infecting a host currently not infected by $i_j$.

Our model assumes the mass action (bi-linear) mechanism of infection^40^. Here we suggest that individuals containing co-infections $i_1,i_2,...,i_k$ can infect healthy or infected hosts in a way that the acquisition of only one new type of parasite is possible at a time, i.e. we neglect simultaneous double, triple, quadruple, etc. infections. In the model equations, $\beta _i$ is the transmission coefficient of the infection by host individuals having only a single type of parasite ($I_i$), whereas $\beta _{i_1,i_2,...,i_k}^{i_j}$, $i_j\in (i_1,i_2,...,i_k)$ denote the transmission coefficient of the parasite of type $i_j$ from an infected host $I_{i_1,i_2,...,i_k}$ to a healthy host or to a host which does not contain the parasite of type $i_j$. The parameters $\alpha _{i_1,i_2,..,i_{k}}$ denote the virulence (i.e. an extra mortality of the host) due to the presence of parasites of types $i_1,i_2,..,i_{k}$. Following previous studies^14,25^, we assume a trade-off between the virulence and the transmission rate (details provided below). Note that in the above model, we consider parasites to be obligate ones; in most cases, we assume that parasites are pathogens, however, the generic nature of the model allows its application to non-pathogenic types of parasites.

In the equation for *S*, *F*(*N*) is the host’s per capita reproduction rate (*N* denotes the total number of hosts). Here assume that *F*(*N*) is a decreasing linear function. $\mu$ is the background (unrelated to parasitism) mortality of the host. For simplicity, we disregard the possible recovery of infected hosts, the corresponding extension of the model can be easily done (e.g. see^25,38^). A summary of the dynamical variables, functions and model parameters is in Table 1. For the particular cases with double, triple and quadruple infections, i.e. for $n=1,2,3,4$, the corresponding explicit equations are provided, for simplicity, in the supplementary material.

**Table 1:** Definitions of variables, parameters and functions used in the model defined by Eqs. (1–4).

Model Component	Meaning	Definition and Values Used
*S*	Density of susceptible (parasitized) hosts	–
$I_{i_1,i_2,...,i_k}$	Density of hosts infected by parasites of types $i_1,i_2,...,i_k$	–
*F*(*N*)	Density-dependent birth rate of susceptible hosts (*N* is the total host density)	$F(N)=r(1-N/K)$
*r*	Maximal per capita reproduction rate of susceptible host	$r=5$
*K*	Carrying capacity of the host population	$K=6$
$\mu$	Natural host density-independent mortality due to factors other than infection	$\mu =0.1$
$\alpha _i$	Virulence of single infected hosts by parasites of type *i*	$\alpha _i=0.2$
$\alpha _{i_1,i_2,...,i_k}$	Virulence of hosts infected by parasites of types $i_1,i_2,...,i_k$	$\alpha _{i_1,i_2,...,i_k}=\sum _{k\in (i_1,i_2,...,i_k)}\alpha _k$
$\beta ^{i_j}_{i_1,i_2,...,i_k}=\frac{\beta _{0i}\alpha _{i}}{K_{0i}+A^{i_j}_{i_1,i_2,...,i_k}}$	Trade-off between the transmission and the effective virulence $A_{i_1,i_2,...,i_k}$ for parasites of type $i_j$, when the host is co-infected by types $i_1,i_2,...,i_k$ parasites	$\beta _{0i}=0.4$, $K_{0i}=0.1$
$A^{i_j}_{i_1,i_2,..,i_k}=\sum _{k\in (i_1,i_2,...,i_k)}C_k\alpha _k$	Effective virulence used in the expression $\beta ^{i_j}_{i_1,i_2,...,i_k}=\frac{\beta _{0i}\alpha _{i}}{K_{0}+A^{i_j}_{i_1,i_2..,i_k}}$	$0\le C_k\le 1$,$C_{i_j}=1$

### Parameterisation of model terms

We implement the standard assumption about the existence of a trade-off between virulence and transmission of pathogens^25,38^. In particular, we will use the following well-known hyperbolic parameterisation to describe the trade-off between transmission and virulence for a single infection of any type *i*

6 $$\begin{aligned} \begin{aligned} \beta ^{i_1}_{i_1}=\frac{\beta _{0i}\alpha _{i_1}}{K_{0i}+\alpha _{i_1}}, \end{aligned} \end{aligned}$$

where $\beta _{0i}$ and $K_{0i}$ are constants (for simplicity, in most cases we assume them to be independent of *i*). Parameterisation of the trade-off function in the case of co-infection is a more complicated matter since this should include mutual interactions between different types of pathogens competing for the same host. To the best of our knowledge, there is no universally accepted way of parametrising $\beta ^{i_j}_{i_1,i_2,...,i_k}$. Here we will use the following expression using a hyperbolic parameterisation

7 $$\begin{aligned} \begin{aligned} \beta ^{i_j}_{i_1,i_2,...,i_k}=\frac{\beta _{0i}\alpha _{j_i}}{K_{0i}+A^{i_j}_{i_1,i_2,...,i_k}}, \end{aligned} \end{aligned}$$

where $A^{i_j}_{i_1,i_2,...,i_k}$ has the meaning of an effective virulence, i.e. a certain function of single-pathogen virulence $\alpha _i$, which describes the presence of all co-infecting pathogens in $I_{i_1,i_2,...,i_k}$ in the ability to transmit pathogen $i_j$ (we assume that $i_j \in ( i_1,i_2,...,i_k)$). For simplicity, we consider the following linear combination of $\alpha _i$

8 $$\begin{aligned} \begin{aligned} A^{i_j}_{i_1,i_2,...,i_k}=\sum _{k\in (i_1,i_2,...,i_k)}C_k\alpha _k{.} \end{aligned} \end{aligned}$$

Note that in the particular case with $C_k=0$ (and $C_{i_j}=1$) we obtain $\beta ^{i_j}_{i_1,i_2,...,i_k}=\frac{\beta _{0i}\alpha _{j_i}}{K_{0}+\alpha _{j_i}}$, which coincide with the same expression as in^25,38^ which was suggested to take place in the absence of competition between the pathogens inside the host and without phenotypic plasticity. However, we should stress that even without a direct competition of pathogens for resources inside the host, co-infections can still affect the transmission of pathogens, for example, by reducing the contact rate of hosts. For the mentioned reason, we assume that generally all $C_k>1$, even in the absence of competition for host resources: in most of our simulations, we considered $C_k=1$. However, for comparison purposes, we also explored the scenario with $C_k=0$, $C_{i_j}=1$.

The parameterisation of the virulence for multiple infections is given by

9 $$\begin{aligned} \begin{aligned} \alpha _{i_1,i_2,...,i_k}=\sum _{k\in (i_1,i_2,...,i_k)}\alpha _k. \end{aligned} \end{aligned}$$

Note that the above expression describes the scenario of the absence of competition for host resources^25^: for a more general case, one needs to introduce weights in the above expression. In this paper, for the sake of simplicity, we do not include explicit competition for host resources which should be done elsewhere.

In this study, we explore long-term evolutionary dynamics using the Adaptive Dynamics framework, which considers ecological (epidemiological) time scales to be much faster than the slow evolutionary dynamics^13,33,41^. The essence of the method employs game theory by the introduction of a rare mutant with slightly different traits into the resident population at ecological equilibrium, or generally, an ecological attractor. This process occurs iteratively, with successive successful mutant invasions excluding the resident^41–44^. Following numerous such invasions and substitutions, the species evolve towards an evolutionary (and convergent) stable singular point called an Evolutionarily Stable Strategy (ESS). Similarly, simultaneous co-evolution of life traits of several pathogens results in a co-Evolutionarily Stable Strategy (co-ESS). The invasion fitness characterises the initial exponential growth (or decay) of a rare mutant and can be utilised in the analytical derivation of an ESS strategy: the evolutionarily singular point signifies the vanishing of the gradient of invasion fitness. We should stress, however, that for the number of co-infections $n>2$, analytical expressions for invasion fitness become intractable, thus we use direct numerical simulations, where we successively introduce rare mutants after the system reaches the close vicinity of its ecological equilibrium. We apply a superinfection framework for mutants and the resident strains of the same type of pathogen^38^, i.e. different strains of the same pathogen cannot coexist in the same host. The equations defining invasion fitness for $n=1,2$ and the corresponding flowcharts are provided in the supplementary material.

### Measuring success of biological control

We need a measure of the success of biological control when using co-infections: we recall that the original goal is to suppress, as much as possible, the target host (pest) species by introducing co-infecting pathogens. The simplest measure of biological control by pathogens is the total number of individuals *N* of the host, which is expected to decrease as a result of control. However, this does not account for the effects of debilitation of the host due to infections. Indeed, a heavily infected pest generally produces considerably less damage to the ecosystem (e.g. less consuming of other species) compared to a healthy pest individual. Reduction in the fitness of the host caused by parasites should arguably be a certain function of infection load related to the virulence, which generally increases in the case of co-infections. As such, the damage of infected individuals $I_{i_1,i_2,...,i_n}$ of the considered pest species on the environment should include some weighting. By following the logic above, we can use the efficient population size of the pest $N_e$ defined as follows as a proxy for the success of biological control

10 $$\begin{aligned} \begin{aligned}{}&N_e=S+\sum _{m=1}^{n}I_m w(\alpha _m)+\sum _{m_1=1}^{n}\sum _{m_2=m_1+1}^{n}I_{m_1,m_2} w(\alpha _{m_1,m_2})\\&\quad +...+\sum _{m_1=1}^{n}\sum _{m_2=m_1+1}^{n}...\sum _{m_k=m_{k-1}+1}^{n}I_{m_1,m_2,...,m_k} w(\alpha _{m_1,m_2,...,m_k})+...+ I_{1,2,...,n}w(\alpha _{1,2,...,n}), \end{aligned} \end{aligned}$$

where $w(\alpha )$ are weighting functions. These functions should be non-linear and have the following properties: $w(0)=1$ (in the absence of infection, the damage is maximal and given by 1), and for $\alpha \gg 1$, we have $w(\alpha )\approx 0$ (i.e. a heavily infected pest produces almost no damage to the environment). Various non-linear functions $w(\alpha )$ satisfy the required above properties. In this study, we consider the following parameterisations of the weighting functions $w(\alpha )$

11 $$\begin{aligned}{}&\begin{aligned} w_1(\alpha )=\frac{a}{1+\exp (b(\alpha -\alpha _0))}, \end{aligned} \end{aligned}$$

12 $$\begin{aligned}{}&\begin{aligned} w_2(\alpha )= \exp (-c\alpha ). \end{aligned} \end{aligned}$$

The above functions are the sigmoidal and the exponential dependencies, respectively; $a,b,c,\alpha _0$ are positive parameters (note that the parameter *a* is not independent, it can be found from the condition $w_1(0)$=1). According to the scenario described by the sigmoidal function, the impact of the infected individuals on the environment is close to that of the healthy ones until the virulence attains some threshold value determined by $\alpha _0$: with the virulence higher than $\alpha _0$, the infected organism becomes too debilitated and its negative impact on the other species and the environment is close to zero. For the scenario modelled by $w_2(\alpha )$, the negative impact on the environment caused by an infected pest exponentially gradually drops with an increase of virulence starting already from low values of $\alpha$. Note that both expressions can describe the particular case, where only healthy individuals of the host would produce damage to the environment, in this case, we constrain the coefficients such that $b,c\gg 1$ and $\alpha _0\ll 1$.

In this study, we also perform a simple cost-effectiveness analysis of biological control with co-infections. Here we apply two well-known cost-effectiveness metrics^45^, the incremental and average cost-effectiveness ratios, denoted by ICER and ACER, respectively. The mathematical expressions for the ICER and the ACER are as follows

13 $$\begin{aligned}{}&\begin{aligned} ICER=\frac{\Delta C}{\Delta B}, \end{aligned} \end{aligned}$$

14 $$\begin{aligned}{}&\begin{aligned} ACER=\frac{C}{B}, \end{aligned} \end{aligned}$$

where $\Delta C$ is the difference between two control strategies to be compared, $\Delta B$ is the difference between the corresponding effectiveness, *C* is the cost of a single control strategy, and *B* is the effectiveness of the considered strategy. For simplicity, we consider the scenario where the cost of biological control of introducing a natural enemy of each type is the same (given by $C_0$, thus adding a new type of enemy to the existing ones results in an extra cost $\Delta C=C_0$). The cost is additive, i.e. implementation of *n* types of enemies requires the cost of $C=nC_0$, we can always assume that $C_0=1$. We must stress that there can be other scenarios, with a non-additive cost of natural enemies, for example, in the situation where the cost of fieldwork to release natural enemies does not largely depend on the total number of enemies used, and the cost of breeding of natural enemies is low. Such cases, however, need to be assessed in some separate study.

To measure the effectiveness of control *B*, we use the normalised efficient population size $N_e(n)$ for *n* co-infections, namely, $B=1-\frac{N_e(n)}{N_e(0)}$. Here $N_e(0)$ denotes the pest density without biological control ($n=0$). The rationale behind this formulation is that the reduction in the negative impact of pests after applying the biological control is arguably proportional to the difference between the numbers $N_e(0)$ and $N_e(n)$, in particular, in the case $N_e(n) \approx 0$ we have $B \approx 1$, i.e. the maximal effectiveness. For the incremental effectiveness -while adding a new parasite to the existing $n-1$ types- we have $\Delta B =\frac{N_e(n-1)-N_e(n)}{N_e(0)}$.

## Results

### Co-existence of multiple parasites at co-ESS

The first step in exploring the model is an investigation of the possibility of coexistence of co-infecting parasites, i.e. whether all introduced types of parasites will eventually survive. We should stress, however, that an exhaustive investigation of the model of an arbitrary *n* and within a wide range of model parameters, including those of the trade-off functions $\beta ^{i_j}_{i_1,i_2,...,i_k}$, is a challenging task and should be done properly elsewhere. We have conducted numerical simulations of the model for a large number of randomly chosen parameter sets. Here we focus on the scenario with the pathogens having similar life-history traits, i.e. close values of $K_{i0},\beta _{0i}$: this allows us to properly compare outcomes obtained for increasing *n*. We stress that despite the closeness of life-history traits, we consider *n* pathogens to be distinct species. We found that for most of the investigated configurations of parameters, the system allows for a co-evolutionary stable attractor ensuring a stable (i.e. involving non-oscillatory population dynamics) coexistence of all types of parasites, provided that the starting values for the evolution of virulence is the ESS strategy obtained for a single-pathogen infection. Note that for $n=1$, the ESS $\alpha$ is given by $\alpha ^*=\sqrt{K_{0i}\mu }$ (see supplementary material SM1.1 for detail).

**Figure 1:**
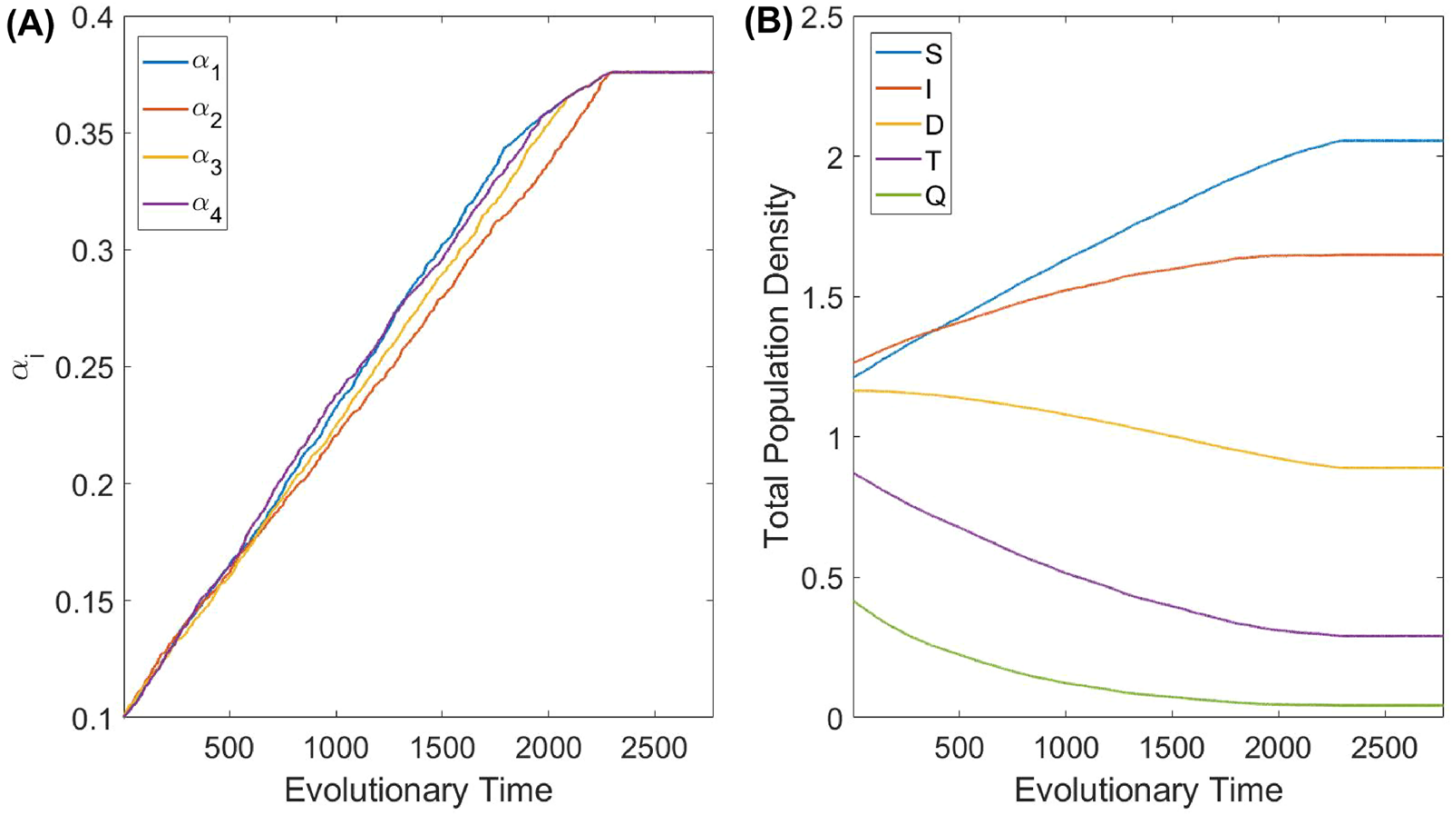
(**A**) Simulated co-evolution of the virulence $\alpha _i$ of parasite type *i*, $i=1,2,3,4$ (the total number of parasite types $n=4$). The transmission rate is parameterised as $\beta ^{i_j}_{i_1,i_2,...,i_k}=\frac{\beta _{0i}\alpha _{j_i}}{K_{0}+\Sigma \alpha _{i}}$. The co-evolution is governed by the surviving strains subsequent to the long-term simulations following the invasion by rare nearby mutants produced by all types of parasites. (**B**) Evolutionary variation of the densities of the healthy host *S*, the host infected by a single parasite *I*, and the host having 2, 3, and 4 co-infections, denoted by *D*, *T*, and *Q*, respectively. In each graph, the horizontal axis measures the evolutionary time, which counts each event of the introduction of a new set of mutants into the system: at each evolutionary time moment the plotted densities are stationary densities on the ecological time scale. All model parameters are as defined in Table 1.

A typical pattern of a joint co-evolution of virulence in the system in a multi-parasite setting with $n=4$ is shown in Fig. 1, constructed for parasites with identical life-history traits (we find that small perturbations of life trait parameters do not affect the results dramatically). In the figure, we also show the changes in the density of healthy hosts as well as densities of hosts (co-)infected by $k=1,2,3,4$ types of parasites through the course of evolution, denoted by *I*, *D*, *T*,  and *Q*, respectively. The figure shows an eventual stable co-ESS of all four strains introduced in the system, where the evolution starts from individual ESS single infection virulence given by $\alpha ^*=\sqrt{K_{0i}\mu }$. One can see that long-term co-evolution results in a reduction of the densities of the host with co-infections. Note that in the multi-dimensional parameter space of virulence $(\alpha _1,\alpha _2,...,\alpha _n)$, a randomly chosen point characterised by a large sum of $\alpha _i$ would most certainly result in the extinction of a co-infected host. Thus, the observed stable co-existence of all types of parasites in our simulations is explained by the fact that the evolutionary trajectory starting from low $\alpha _i$ avoids domains of extinction. On the other hand, starting co-evolution with some large values of virulence may result in an evolutionary suicide (see *Evolutionary suicide in the model with co-infections* section for detail).

**Figure 2:**
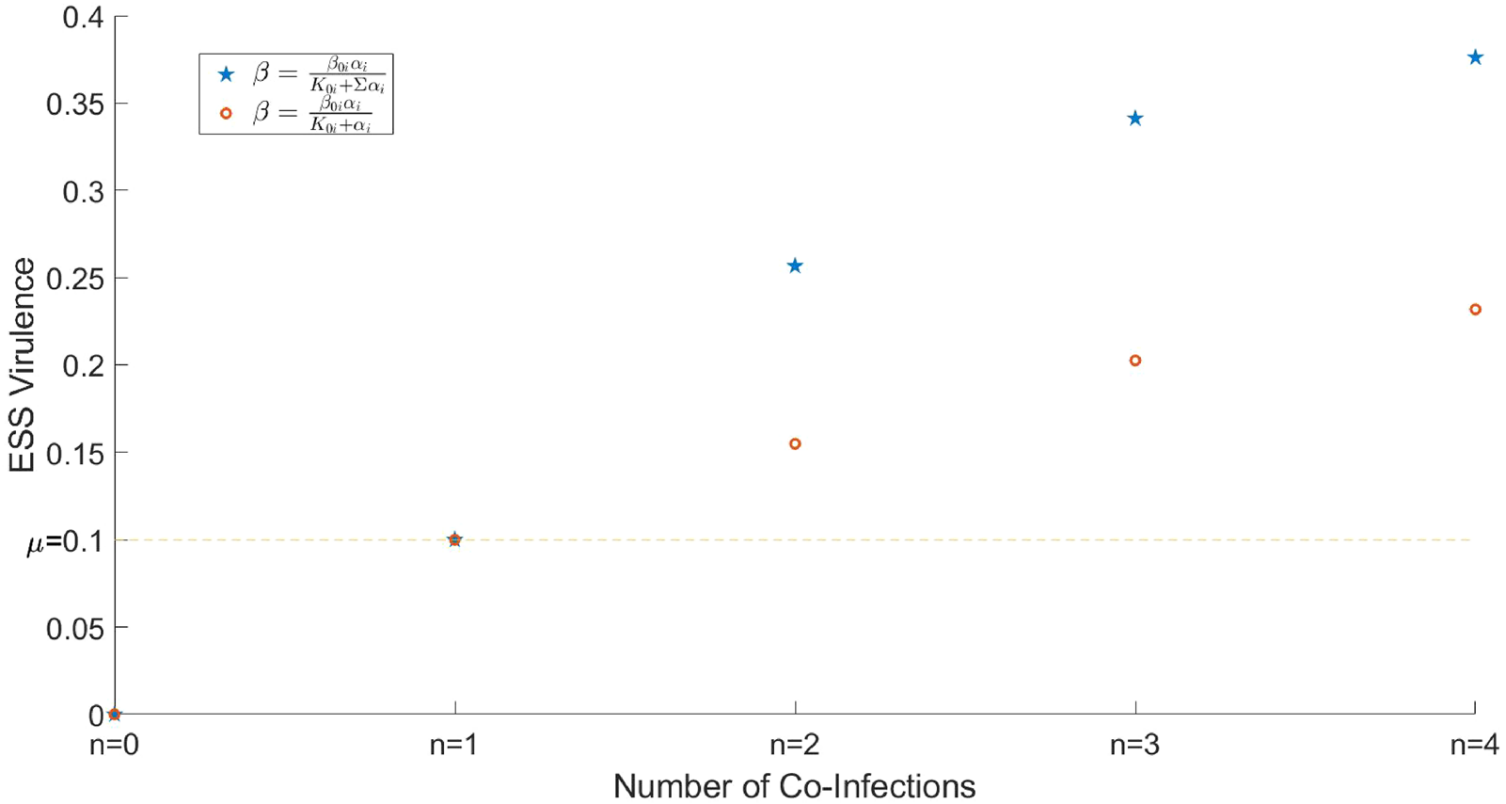
Dependence of co-Evolutionary Stable Virulence (co-ESS Virulence) on the number of co-infections in the system. The blue stars represent the co-ESS virulence in the case where the effective virulence is the sum of all $\alpha _i$ (transmission rate given by $\beta ^{i_j}_{i_1,i_2,...,i_k}=\frac{\beta _{0i}\alpha _{j_i}}{K_{0}+\Sigma \alpha _{i}}$), and the orange circles show co-ESS virulence for the particular case with only $C_{i_j}=1$ (transmission rate given by $\beta ^{i_j}_{i_1,i_2,...,i_k}=\frac{\beta _{0i}\alpha _{j_i}}{K_{0}+\alpha _{j_i}}$). The horizontal dashed line denotes the ESS virulence for a single infection case, i.e. in the absence of co-infections. All model parameters are as defined in Table 1.

We investigated the dependence of the co-ESS virulence on the maximal number of co-infections *n*. In the case, where all $K_{i0},\beta _{0i}$ are identical, the graph for the resultant co-ESS $\alpha _i$ is shown in Fig. 2 constructed for two different scenarios of parameterisation of the transmission rate: $\beta ^{i_j}_{i_1,i_2,...,i_k}=\frac{\beta _{0i}\alpha _{j_i}}{K_{0}+\Sigma \alpha _{i}}$ and $\beta ^{i_j}_{i_1,i_2,...,i_k}=\frac{\beta _{0i}\alpha _{j_i}}{K_{0}+\alpha _{j_i}}$. One can see that for both scenarios the co-ESS virulence increases with the number of co-infections. In particular, in the presence of multiple parasites, the resultant evolutionary virulence is always higher that in the case of a single infection. On the other hand, the growth in virulence with *n* is decelerating, for example, in scenario with $\beta ^{i_j}_{i_1,i_2,...,i_k}=\frac{\beta _{0i}\alpha _{j_i}}{K_{0}+\Sigma \alpha _{i}}$ transition from $n=1$ to $n=2$ results in an increase of virulence by more than $150\%$, whereas transition from $n=3$ to $n=4$ leads to a much smaller increase of less than $10\%$ (for the scenario with $\beta ^{i_j}_{i_1,i_2,...,i_k}=\frac{\beta _{0i}\alpha _{j_i}}{K_{0}+\alpha _{j_i}}$ increase in virulence is even smaller). Considering a higher number of co-infections results in an only marginal increase in virulence. We found that similar observations hold for the other checked sets of models parameters.

### Impact of the number of co-infections on success of biological control

**Figure 3:**
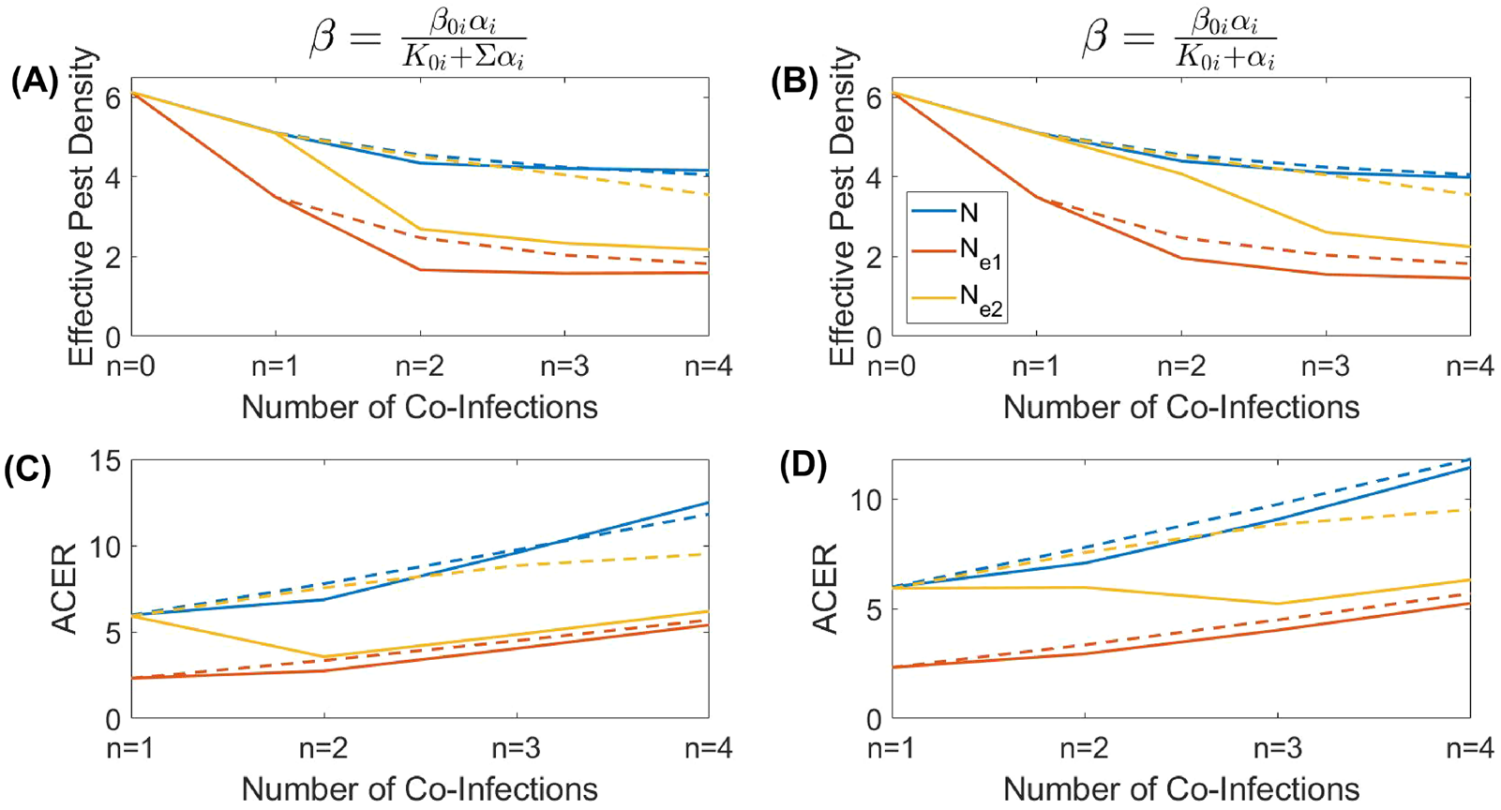
Dependence of (**A**, **B**) the effective pest density and (**C**, **D**) the ACER on the possible number of co-infections for two cases of transmission $\beta ^{i_j}_{i_1,i_2,...,i_k}$. The blue curves display the total species density *N* at the co-ESS virulence, the orange and yellow curves display the efficient population size of the pest $N_e$ at the ESS virulence when weighting functions $w_1$ and $w_2$ are used, given by (11–12) respectively. The dashed curves correspond to the short-time scale scenario, with the virulence given by the ESS for a single infection ($n=1$). Within the weighting functions $w_1$ and $w_2$, the parameters are as follows; $\alpha _0=0.4$, $a=1$, $b=20$ and $c=5$, all other model parameters are as defined in Table 1.

We explored the outcome of biological control as a function of the number of parasites co-infecting the target host species. We considered two different scenarios of biological control based on a long-term and a short-term evolution of virulence. In the former case, we assume that the virulence of each parasite is given by its co-ESS value: in Fig. 1 this corresponds to large evolutionary times. In the latter scenario, we consider that over a short time period the virulence of each parasite remains unchanged and is equal to its initial value (which is assumed to be the ESS virulence for a single infection $\alpha ^*=\sqrt{K_{0i}\mu }$): in the example, in Fig. 1 this is the starting point of the evolution. The outcomes of biological control for both scenarios and for the two different parameterisations of $\beta ^{i_j}_{i_1,i_2,...,i_k}$ are shown in Fig. 3 (the upper panel), where we plot the total density of host as well as its effective pest densities defined by Eq. (10) with the weighting functions given by Eqs. (12) and (11).

One can see from the figure that in most cases, adding co-infecting parasites nominally reduces the negative impact of the target pest on the environment measured by indicators *N*, $N_{ei}$. However, adding new parasites does not largely amend the control of pests beyond the results obtained for the simplest co-infection scenario with $n=2$. For example, the total density of host *N* only marginally decreases starting from the double infection setting. A similar conclusion is made using the indicator $N_{e2}$. On the other hand, depending of parameterisation of $\beta ^{i_j}_{i_1,i_2,...,i_k}$ the value of $N_{e1}$ can show a growth for the transition from a double infection to triple or quadruple co-infection settings (Fig. 3A). In other words, surprisingly, biological control becomes less efficient when using more enemies. A comparison between short-term and long-term outcomes of biological control (depicted by solid and dashed lines, respectively) shows that in the majority of cases on a short-term timescale, biological control is less efficient than the one expected to be based on long-term evolution. This is related to the fact that the ESS virulence for a single infection is less than that under multiple infection settings (see Fig. 2).

Our cost-effectiveness analysis demonstrates (see Fig. 3, the bottom panel) that for most scenarios the ACER increases with the number of co-infections, except in the case with $N_{e2}$ measured at ESS. An increase in the ACER with *n* signifies that adding new types of natural enemies becomes inefficient, so implementing a single parasite would be more beneficial (we remind that we use the assumption on the additive of cost of implementing natural enemies). In the case where efficient density is measured by $N_{e2}$, the optimal control requires co-infections, however, even in this situation, the cost-efficient control is achievable for only a few co-infections ($n=2,3$). Similar conclusions can be made using the second metric of cost-effectiveness, the ICER, see SM5 for the corresponding graphs.

**Figure 4:**
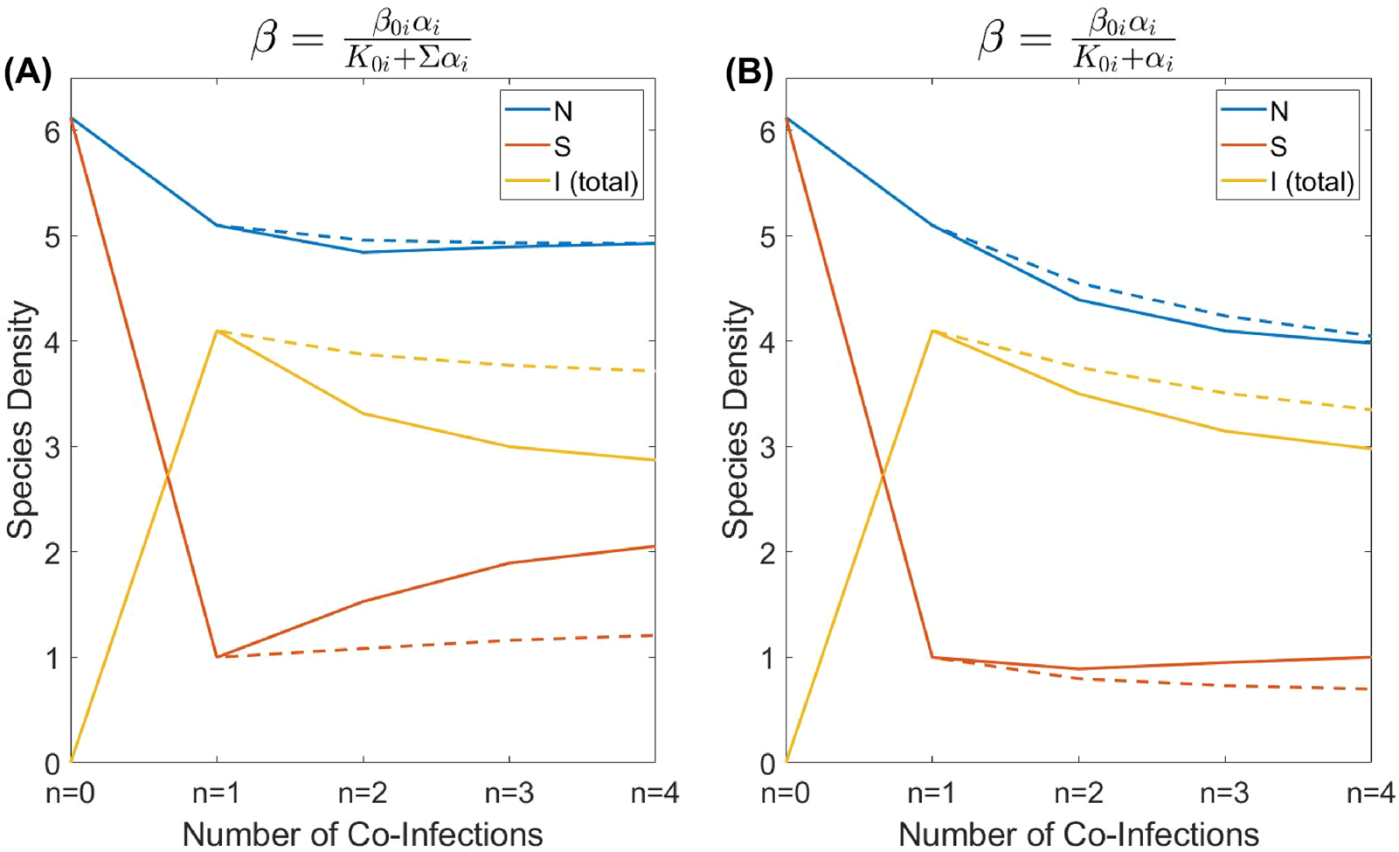
Dependence of the species density on the possible number of co-infections for two cases of transmission $\beta ^{i_j}_{i_1,i_2,...,i_k}$. The blue curves display the total species density *N* at the ESS virulence, orange curves represent the density of susceptible hosts *S* at the ESS virulence, and yellow curves display the total density of infected hosts *I* at the ESS virulence. The dashed curves correspond to the short-time scale scenario, with the virulence given by the ESS for a single infection ($n=1$). All model parameters are as defined in Table 1.

Figure 4 reveals the densities of the susceptible and infected hosts (regardless of the number of co-infections per host) corresponding to the values of virulence shown in Fig. 2. An increase in the number of co-infections reduces the density of infected hosts, whereas the density of healthy hosts *S* increases already starting from $n=2$. An increase in *S* is especially pronounced for the transmission rate $\beta ^{i_j}_{i_1,i_2,...,i_k}=\frac{\beta _{0i}\alpha _{j_i}}{K_{0}+\Sigma \alpha _{i}}$ (left panel A). Under the ecological scenario, where susceptible hosts mostly damage the ecosystem and pest individuals containing parasites are harmless ($b,c\gg 1$ in the corresponding weighting functions $w_{1,2}(\alpha )$), using several parasites ($n>1$) instead of a single one would impede the biological control. Similar conclusion can be formally made using the cost-effectiveness analysis, where we measure the effectiveness of control is based on the density of healthy host *S* (see SM5 for detail). Another interesting observation is that for multiple infections, a long-term evolution would eventually result in a rise in the density of the susceptible hosts. Thus, under the mentioned scenario where most damage to the ecosystem is caused by *S*, the use of multiple parasites in pest management will only produce a temporary positive solution.

**Figure 5:**
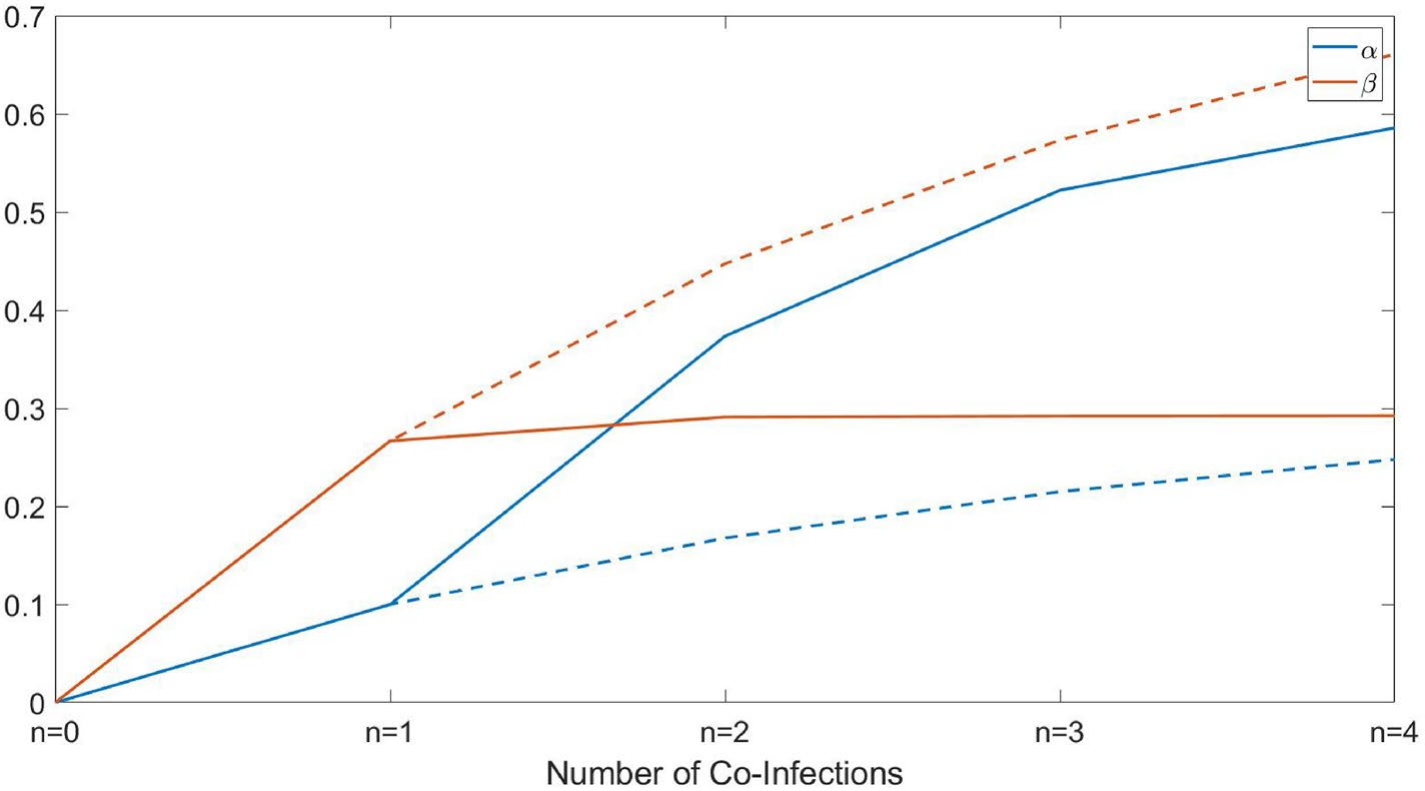
Dependence of the average virulence and transmission on the possible number of co-infections. The blue curves display the average virulence of the population at the ESS virulence, and the orange curves represent the average transmission of the population at the ESS virulence. The solid curves are for the case where the effective virulence is the sum of all $\alpha _i$ ($\beta ^{i_j}_{i_1,i_2,...,i_k}=\frac{\beta _{0i}\alpha _{j_i}}{K_{0}+\Sigma \alpha _{i}}$) and the dashed curves are that but in the particular case with only $C_{i_j}=1$ ($\beta ^{i_j}_{i_1,i_2,...,i_k}=\frac{\beta _{0i}\alpha _{j_i}}{K_{0}+\alpha _{j_i}}$). All model parameters are as defined in Table 1.

Figure 5 presents the co-ESS-based average virulence ${\bar{\alpha }}$ in the system calculated as a weighted sum of $\alpha _{i_1,..,i_k}$ ($k=1,2,...n$) across all infected hosts compartments taking into account their relative abundance. The same figure shows the average transmission rate ${\bar{\beta }}$ while infecting *S* by all possible configurations of infected hosts $I_{i_1,i_2,...,i_n}$ averaged in the same way as the virulence ${\bar{\alpha }}$. As before, we explore two different parameterisations of $\beta ^{i_j}_{i_1,i_2,...,i_k}$. One can see that for the scenario where $\beta ^{i_j}_{i_1,i_2,...,i_k}=\frac{\beta _{0i}\alpha _{j_i}}{K_{0}+\Sigma \alpha _{i}}$, we observe a pronounced increase in ${\bar{\alpha }}(n)$ which is faster than that of ${\bar{\beta }}(n)$. This allows us to understand the increase in the density of *S* with *n* shown in Fig. 4. Indeed, one can easily prove that for the stationary density of *S* we have $S_0=(\mu +{\bar{\alpha }})/{\bar{\beta }}$. For a small background mortality $\mu$, the stationary density of host is mostly determined by the ratio of ${\bar{\alpha }}$ and ${\bar{\beta }}$. As such, when adding parasites species, a fast increase in ${\bar{\alpha }}$ with a slowly changing ${\bar{\beta }}$ results in a raise of the density of *S*. Similar reasoning holds for the other scenario of parameterisation of $\beta ^{i_j}_{i_1,i_2,...,i_k}$, in this case for the stationary *S* we have $S_0=(\mu +\alpha _i)/{\bar{\beta }}$, where $\alpha _i$ is an individual co-ESS virulence.

**Figure 6:**
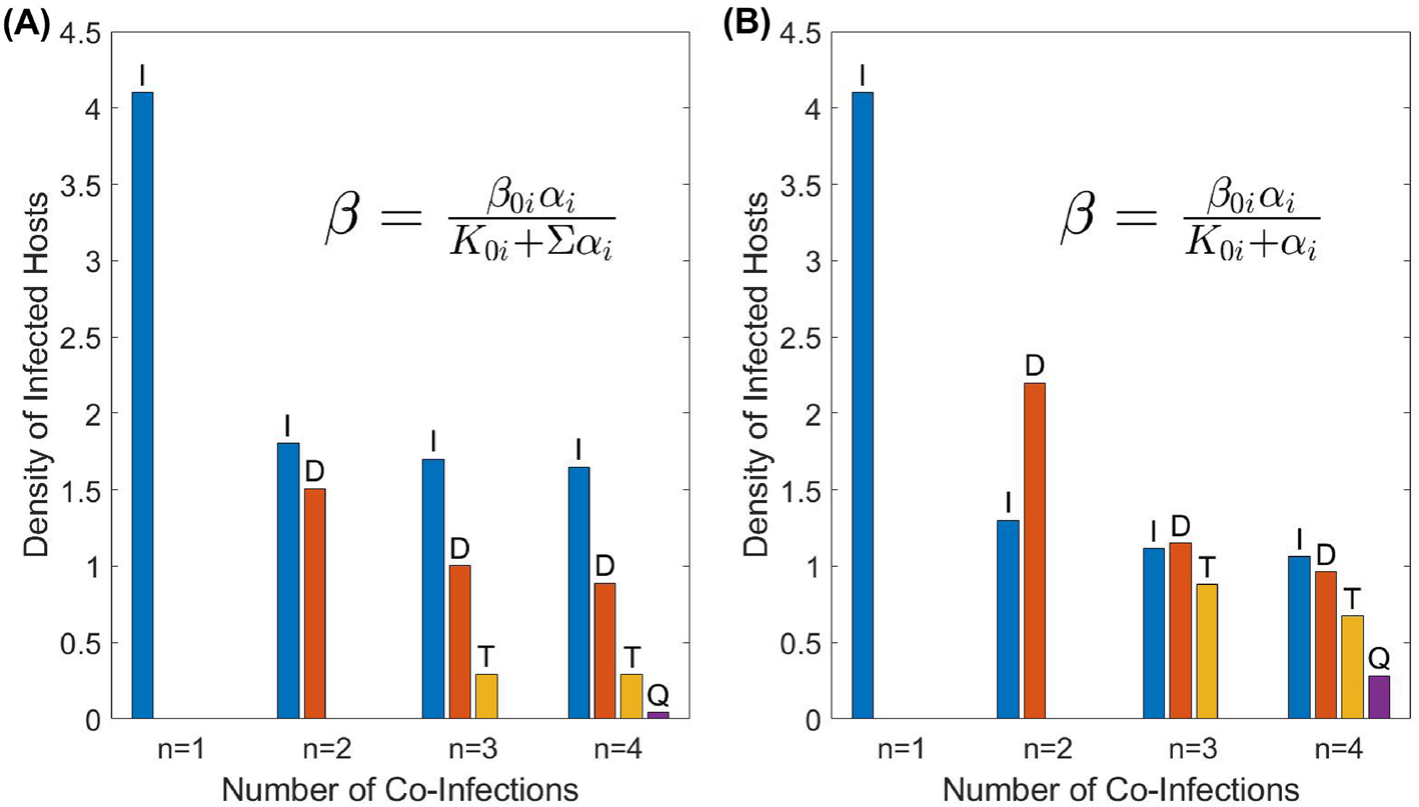
Host densities of possible co-infections for different cases of transmission. The blue bars represent the density of host infected by a single infection (*I*), the orange bars display the density of hosts doubly infected (*D*), the yellow bars show the density of triply infected hosts (*T*), and the purple bars are the density of quadruply infected hosts (*Q*). All model parameters are as defined in Table 1.

The composition of the compartment of infected hosts according to the number of co-infections is shown in Fig. 6. Generally, the population density of co-infected hosts decreases with several simultaneous infections inside the host. For example, the density of infected hosts with quadruple co-infections *Q* is small compared to hosts with a single type infection only (*I*). Our simulations show that the same trend persists for $n>4$ (not shown result). By comparing the right and left panels in Fig. 6, an important conclusion is the interplay of the two following factors: (i) the relative proportion of the type of hosts with a particular type of infection and (ii) the absolute value of virulence that determines the average virulence ${\bar{\alpha }}$. For example, consider $n=2$, although the proportion of doubly infected host *D* is higher for the scenario shown in panel (B), the resultant ${\bar{\alpha }}$ is still higher for the scenario in panel (A) since the corresponding co-ESS virulence $\alpha _i$ is sufficiency larger for (A) (see Fig. 2 for detail). Along with the absolute values of densities of infected hosts, we also evaluated the corresponding prevalence of co-infections for various numbers of parasites’ types, see SM6. For all considered scenarios, we also estimated the probabilities of a newborn healthy host to be infected by all types of parasites, see SM6.

### Evolutionary suicide(s) in the system with co-infections

**Figure 7:**
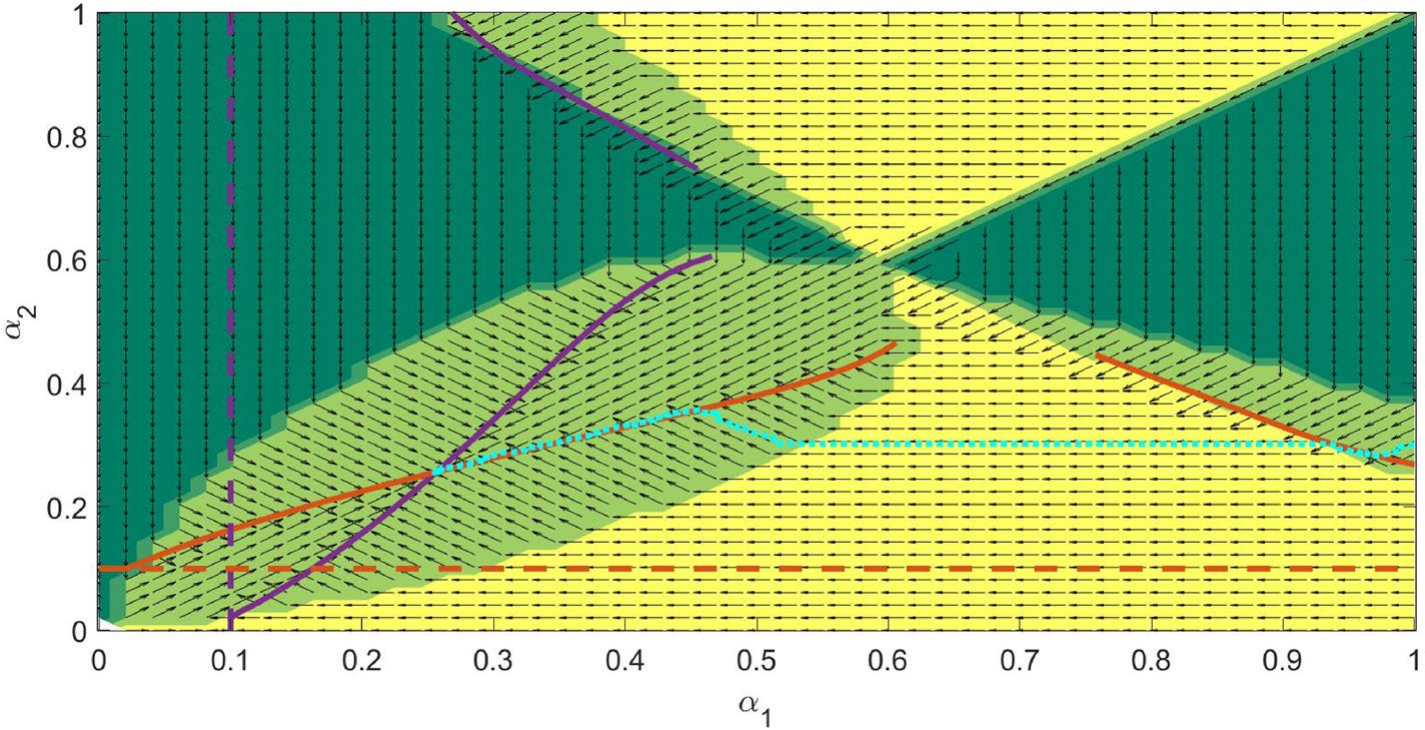
Regions of coexistence across the domain $(\alpha _1,\alpha _2)$ with arrows representing the direction in which evolution is attracted to. The dark green regions represent the domains for which $I_1=0$ but $I_2\ne 0$, the yellow domains represent the domains for which $I_1\ne 0$ but $I_2=0$ and finally, the light green regions represent the coexistence domains ($I_1\ne 0$, $I_2\ne 0$). The solid lines show the dependence of the Evolutionary Stable Strategy ($\alpha _2$) on the virulence $\alpha _S^*$ of the parasite 1 ($I_1$) and vice versa. The dashed lines represent the ESS in the absence of $I_1$ (or $I_2$). The dotted curve represents an example of the evolutionary trajectory with evolutionary suicide (see Fig. 8). All model parameters are as defined in Table 1.

Another interesting outcome of the model is an evolutionary suicide when one or several parasites initially present eventually go extinct over the course of evolution. Our simulations demonstrate the possibility of evolutionary suicide for various $n>1$. For easy visualisation, we present an example of evolutionary suicide with two co-evolving parasites. The mechanism of evolutionary suicide for $n=2$ can be understood from Fig. 7 representing the plane of co-evolving parameters $\alpha _1$ and $\alpha _2$. The purple and red solid curves show the ESS virulence $\alpha _i(\alpha _j)$ where only the parasite *i* can evolve with the other fixed at $\alpha _j$. For example, each point of the red curve gives the ESS virulence for $\alpha _2$ for a fixed non-evolving $\alpha _1$. The intersection of the ESS curves $\alpha _i(\alpha _j)$ ($i,j=1,2, i\ne j$) provides the co-ESS point. The horizontal and vertical dashed straight lines represent the virulence of a single parasite given by $\alpha ^*=\sqrt{K_{0i}\mu }$. Different colours of the domains depict the regions of persistence of a particular parasite or their mutual co-existence as a co-infection (see figure caption for detail). The key condition for having evolutionary suicide is disruption of the curve $\alpha _i(\alpha _j)$, which becomes undefined within some range of $\alpha _j$ (see Fig. 7).

As we mentioned in the *Co-existence of co-infecting parasite at co-ESS* Section, starting a co-evolution trajectory around $\alpha ^*=\sqrt{K_{0i}\mu }$ for each pathogen species causes a monotonic increase of $\alpha _i$ with eventually approaching the co-ESS, in this case, both pathogens will co-exist. However, if the initial choice of $\alpha _i$ is such that for one parasite its virulence is high, co-evolution would involve an evolutionary suicide scenario. This is shown in Fig. 7, where the evolutionary trajectory is denoted by the thickly dotted cyan curve. Starting from a region of co-existence of both parasites, the trajectory gets into the yellow domain, where only parasite $I_1$ can survive since the mortality of $I_2$ and $I_{1,2}$ is too high. In other words, co-evolution of virulence pushes the trajectory to the boundary of extinction of $I_2$. Further evolution of $I_1$ alone results in a reduction of its virulence towards the value of $\alpha ^*=\sqrt{K_{01}\mu }$. However, under a hypothetical scenario of the re-introduction of parasite $I_2$ (e.g. under the biological control framework, one can try re-introducing a second parasite), the co-existence of both parasites becomes possible again at some lower values of $\alpha _i$ located in the large light green domain. A further co-evolution would result in a decrease in $\alpha _i$ towards their co-ESS values.

**Figure 8:**
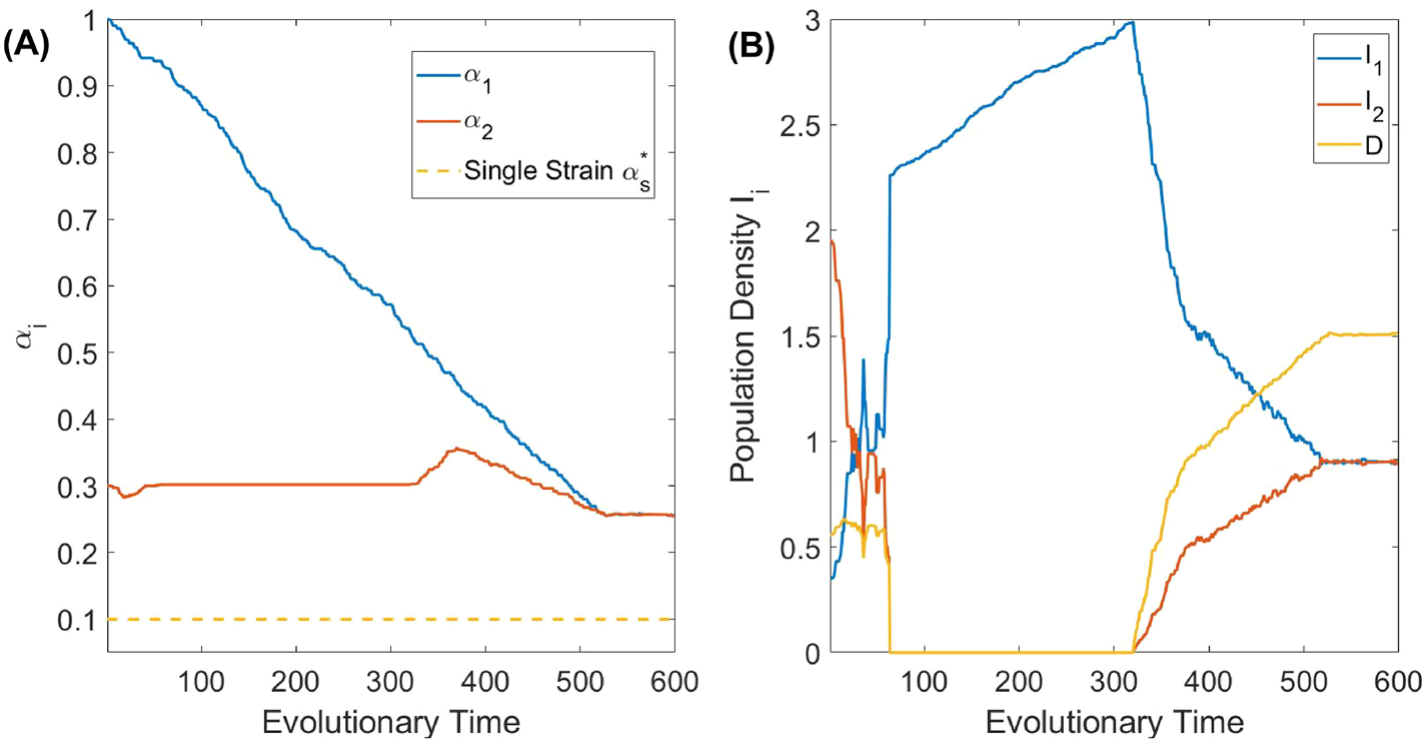
Simulated co-evolution of the virulence of two parasites species, $n=2$. The Adaptive Dynamics based evolution is governed by the surviving strains subsequent to the long-term simulations following the invasion by rare nearby mutants. Panel (**A**) shows the evolution of the virulence of the individual virulence of the two parasites species. Panel (**B**) shows how the densities of the hosts infected by a single parasite $I_i$ and double infected by both parasites *D*, vary as the virulence evolves. All model parameters are as defined in Table 1.

Figure 8 shows the considered above scenario of co-evolutionary suicide with a further re-introduction of extinct species through evolutionary time. In the figure, we show the co-evolution of virulence in panel (A). The stationary densities of infected host $I_{1}, I_{2}$ and $I_{1,2}$, corresponding to the evolving virulence, are shown in panel (B). One can see that without re-introducing the parasite, the evolutionary suicide causes a failure of the originally conceived pest management using both parasites. Note that qualitatively similar behaviour is observed for other numbers of co-infections *n* used in the control of the host.

**Figure 9:**
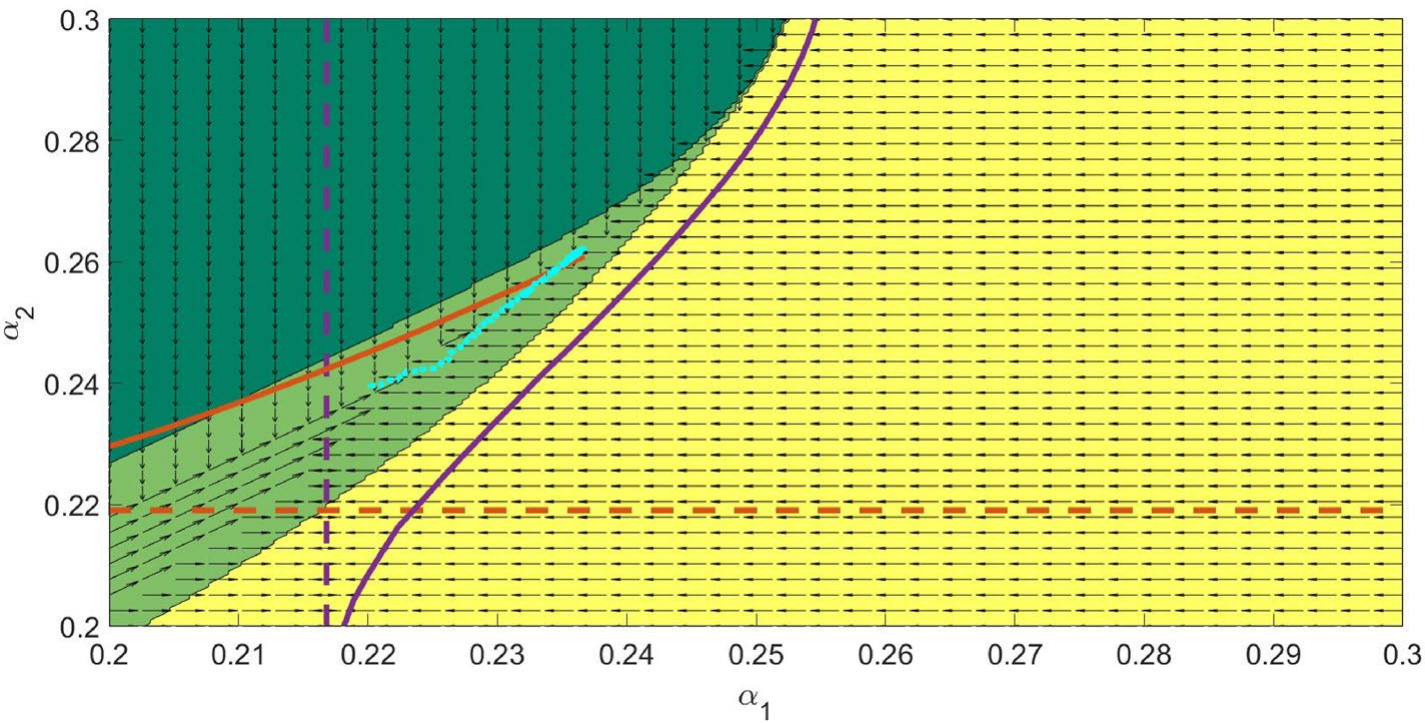
Simulated co-evolution of the virulence of with two co-infections, constructed for the parameters $\beta _{01}=\beta _{02}=0.21$, $K_1=0.47$ and $K_2=0.48$. The dark green regions represent the domains for which $I_1=0$ but $I_2\ne 0$, the yellow domains represent the domains for which $I_1\ne 0$ but $I_2=0$ and finally, the light green regions represent the coexistence domains ($I_1\ne 0$, $I_2\ne 0$). The solid lines show the dependence of the Evolutionary Stable Strategy ($\alpha _2$) on the virulence $\alpha _S^*$ of parasite type 1 ($I_1$) and vice versa. The dashed lines represent the ESS in the absence of $I_1$ (or $I_2$). The dotted curve represents an example of the evolutionary trajectory with evolutionary suicide (see Fig. 10). The Adaptive Dynamics based evolution is governed by the surviving strains subsequent to the long term simulations following the invasion by rare nearby mutants. All other model parameters are as defined in Table 1.

Another type of evolutionary suicide is observed in the case when no co-ESS is feasible in the system. For example, such a scenario can occur for slightly different values of coefficients $K_{0i}$ in the trade-off function of the transmission rate and a higher background mortality $\mu$. For $n=2$, the corresponding the plane of co-evolving parameters $(\alpha _1, \alpha _2)$ is shown in Fig. 9. The notation of the curves and domains in the figure has the same meaning as in Fig. 7. The single ESS curves do not intersect, thus there is no co-ESS point in the system. When the evolutionary trajectory starts from low values, the values of $(\alpha _1, \alpha _2)$ increase and reach the vicinity of the upper sharp corner of the domain of coexistence of co-infecting parasites. The further co-evolution of $\alpha _i$ produces random mutations which land in a domain where only one type of parasite (1 or 2) can persist. The resultant surviving type of parasite (i.e. 1 or 2) will be determined by a particular realisation of a stochastic process of mutations. The other parasite species should go extinct and the surviving parasite would drop to its ESS strategy given by $\alpha ^*=\sqrt{K_{0i}\mu }$ and denoted by a dashed straight line in the figure.

Without re-introducing the extinct parasite species into the system, the co-evolution endpoint would be $\alpha ^*=\sqrt{K_{0i}\mu }$ of the surviving type *i*. However, under realistic ecological settings, a natural re-introduction of the other type of parasite can be possible since (i) the evolutionary and ecological processes can occur on a close time scale (so the total extinction of the other parasite type does not occur before new mutations are generated) and (ii) spatial heterogeneity of the environment can play a role, so local interaction may not be fully synchronised and re-introduction of a previously locally extinct parasite is possible via dispersal from another site. The mentioned scenarios can be mathematically modelled by the introduction of all types *n* of parasites at rare densities at each evolutionary time step. For each evolutionary step, the value of virulence is considered to be given by a small random mutation of the currently present (or previously extinct) parasite strain. Under the mentioned settings, we observe an interesting regime of long-term stochastic co-existence of all *n* types of parasite at the edge of extinction.

**Figure 10:**
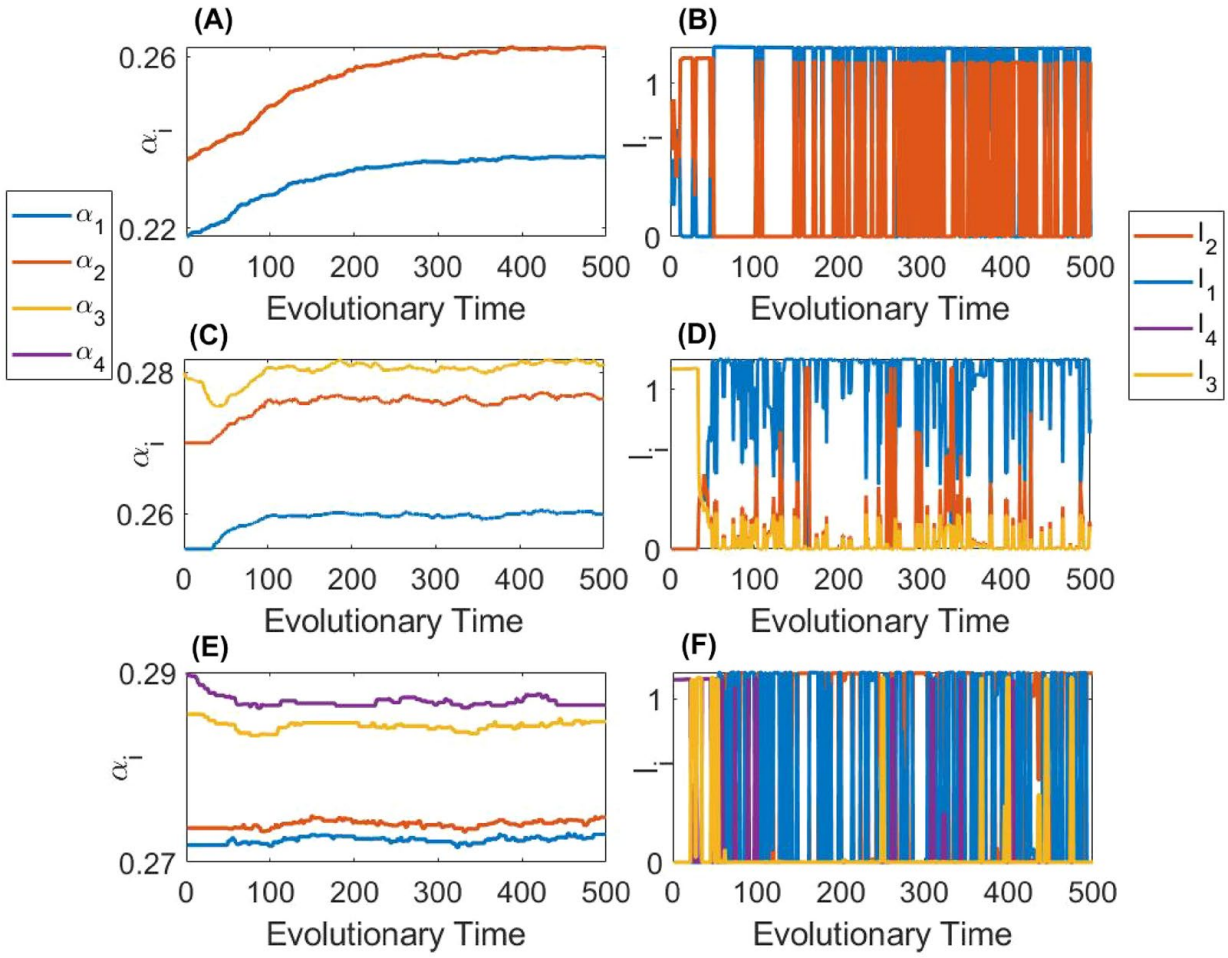
Simulated co-evolution of the virulence of all parasite types constructed for the co-infection number $n=2,3,4$. Evolution is governed by long-term simulations following the invasion of nearby mutants. Panels (**A**), (**C**) and (**E**) show the evolution of the virulence of all individual virulence $\alpha _i$. Panels (**B**), (**D**) and (**F**) show how the densities of the hosts infected by a single strain $I_i$ vary as the virulence evolves. Panels (**A**) and (**B**) consider the case with $n=2$ when $\beta _{01}=\beta _{02}=0.21$, $K_1=0.47$ and $K_2=0.48$. Panels (**C**) and (**D**) consider the case with $n=3$ when $\beta _{01}=\beta _{02}=\beta _{03}=0.21$, $K_1=0.472$, $K_2=0.476$ and $K_3=0.477$. Panels (**E**) and (**F**) consider the case with $n=3$ when $\beta _{01}=\beta _{02}=\beta _{03}=\beta _{04}=0.21$, $K_1=0.473$, $K_2=0.47325$, $K_3=0.4745$ and $K_4=0.47475$. All other model parameters are as defined in Table 1.

Several examples of the co-existence of parasites at the edge of extinction are shown in Fig. 10, plotted for different total possible parasite species in the system ($n=2,3,4$). For each *n*, we show both co-evolution of virulence in the left column as well as the change in species densities of $I_i$ in the right column (for simplicity, we do not show the density of the co-infected host). One can see that the system constantly switches between different dominating species of parasite, where the other parasite species density is close to zero until a new parasite becomes the most abundant one for some time. For the biological control of the pest, the realisation of the given scenario should signify its actual failure. Indeed, despite the presence of multiple parasites in the system (i.e. high biodiversity), the desired joint effect from co-infections cannot be observed due to repeated evolutionary suicides.

## Discussion

Currently, simultaneous implementation of several natural enemies, such as co-infecting parasites (pathogens), to control various pest species is considered to be a promising approach. Among others, there are two major reasons for that. Firstly, empirical studies show that the simultaneous presence of several parasite species inside the host body usually increases the mortality of the target pest species^2,20,25,32^. Secondly, theoretical models predict that a long-term evolution under co-infection settings usually promotes the increase of the virulence of each parasite species, compared to the single-infection scenario^25,26^. The combination of the two pre-mentioned reasons should result in a high increase in the mortality of the pest and as a logical consequence, one would expect more efficient pest management by implementing several parasites rather than a single one. In this study, we critically revisit this apparent common-sense conclusion. The need for such revision is dictated by the fact that despite the abundant literature on co-infections under various scenarios, there is only a very limited connection between the theoretical results to practical pest management and biological control. In particular, here we address the fundamental question about the link between the diversity of parasite community and the negative impact of the host on the ecosystem^32^: we explore how the number of co-infections of effects the biological control of the pest.

The main theoretical findings of this study are the following. Firstly, we find that a gradual increase in the number of co-infections enhances the value of the evolutionary stable virulence of parasites. However, such an increase quickly becomes only marginal when the number of co-infections is larger than $n=2$. Secondly, we show that the efficiency of bio-control, measured via the weighted population density $N_{e}$ of pests, does not essentially drop with an increase in the number of parasites species used. Similar observation comes from our formal cost-effectiveness analysis, which in many cases predicts inefficiency of control by co-infections as compared to a single infection control strategy (note that relaxing our key assumption on the additive cost of control can alter this conclusion). Moreover, under the scenario where only healthy pest individuals cause significant damage to the environment, adding more co-infections becomes counter-productive: the number of susceptible hosts *S* increases with *n* as a consequence of an increase in the overall mortality of the infected host. Thirdly, a decrease in the transmission rate caused by the presence of co-infections generally impedes bio-control (cf. the results obtained for $\beta ^{i_j}_{i_1,i_2,...,i_k}=\frac{\beta _{0i}\alpha _{j_i}}{K_{0}+\Sigma \alpha _{i}}$ and $\beta ^{i_j}_{i_1,i_2,...,i_k}=\frac{\beta _{0i}\alpha _{j_i}}{K_{0}+\alpha _{j_i}}$). Finally, the system can exhibit evolutionary suicide, where long-term evolution leads to the extinction of some parasite species. The latter prediction highlights the importance to consider both long-term and short-term outcomes of biological control of pests separately. Our main conclusion is that the ‘the more enemies, the better’ principle may not always apply to the multi-enemy control of the pest, at least under the current assumptions.

We should stress that apart from the current study, only a small number of models have so far considered a host-parasite model with more than two different types of parasites^32^. For example, May and Nowak in their pioneering study^39^ proposed a co-infection model where hosts can be infected by an arbitrary number of strains. However, two simplifying assumptions were made in the mentioned study: (i) the virulence expressed by co-infected hosts was equal to that of the most virulent parasite and (ii) transmission rates were not affected by the presence of other parasites. Under the above and some other assumptions (e.g. the absence of the transmission-virulence trade-off), the analytical expression for the virulence as a function of *n* was obtained. Interestingly, although theoretical settings were different, the obtained analytical expression for the overall virulence also shows a quick saturation with *n*, emphasising the generality of our findings. Another model with an arbitrary number of co-infections was developed by S. Lion^46^, however, unlike our model, the proposed framework mostly focused on the kin-selection and did not consider parasites as distinct species. However, in the model with a kin selection, a quick saturation of virulence as a function of the number of parasites is still observed, confirming the generality of our results for multi-species infection models.

An important practical application of this theoretical study can be the biological control of the red palm weevil, *Rhynchophorus ferrugineus*, which is the main devastating pest of date palms in the Kingdom of Saudi Arabia and across the globe. Chemical control of the pest is challenging due to the cryptic nature of the insect^47^. Recent studies suggest that simultaneous implementation of several enemies would amend the control of the pest, in particular, it would reduce the population numbers of the insect. For example, a promising approach would be a joint use of entomopathogenic nematodes and entomopathogenic fungi, for example species *Beauveria bassiana* and *Metarhizium anisopliae*^2,48^. Importantly, all mentioned control agents are obligate parasites; they do not present any danger to non-target organisms or the environment. It was also reported that joint use of entomopathogenic fungi and entomopathogenic nematodes has an additive effect on the pest mortality^1,2^. Similar effects between entomopathogenic nematodes and fungi were observed in biological control of other insect pests devastating orchards such as the black vine weevil^24^. These experimental results are considered to be promising since they reveal high mortality in the co-infected host. On the other hand, our model highlights the importance of the transmission rate by insects jointly infected by fungi and nematodes, which to our best knowledge, remains largely understudied. Interestingly, some preliminary experimental studies indicate the overall reduction of fitness of the red palm weevil under multiple infections^2^, which can signify a slower movement of adult insects, thus the transmission rate of pathogens would be arguably described by the parameterisation $\beta ^{i_j}_{i_1,i_2,...,i_k}=\frac{\beta _{0i}\alpha _{j_i}}{K_{0}+\Sigma \alpha _{i}}$. In this case, the model predicts an only marginal improvement of biological control of the pest or even greater damage to the palm trees since the number of healthy hosts is predicted to increase. From the pest management perspective, one would need to apply a different control strategy, which is distinct from the natural control, for example, via frequent artificial releases of natural enemies to push the system away from its evolutionary equilibrium. Our study highlights the need for a shift from experimental studies of the virulence of the red palm weevil to a comprehensive investigation of the transmission of the mode of entomopathogenic fungi and nematodes under the co-infection scenario.

Another ecological application of the current study can be understanding the effects of co-infection of the host by distinct viral strains: in this case, *n* can be large. This can be, for example, the biological control of the chestnut blight disease by a hypovirus^8,49^ in the United States and Europe. The chestnut blight disease is caused by a fungus with several distinct viral strains used to infect the pest, promoting biological control^49,50^. However, despite intensive efforts, biological control of the chestnut blight disease in the United States has not yet been successful, with a large number of trees being killed. The other important ecological case of multi-viral infection includes yellow dwarf viruses. Yellow dwarf viruses show high diversity and can infect several agricultural and natural grass species. Unlike hypoviruses fighting the chestnut blight, yellow dwarf viruses are considered a nuisance. In both pre-mentioned cases, regardless of the ecological interpretation of viral pathogens (positive or negative), having high biodiversity of co-infections would have a saturated effect on the reduction of the population numbers of the host.

The system allowing for co-infections demonstrates the possibility of evolutionary suicide, which was not reported in similar models earlier (even for the number of co-infecting parasites $n=2$). The occurrence of evolutionary suicide under various scenarios is currently a growing area of research in evolution and ecology, although it is mostly based on theoretical predictions^51–54^. The simplest scenario of evolutionary suicide is shown in Fig. 8, where co-evolution of virulence competing for the same host eventually leads to the extinction of certain pathogens initially introduced to the system. However, further re-introduction of the extinct pathogen strain at a later time should guarantee an eventual evolutionary stable co-existence of all pathogens (see Fig. 8). From the biological control perspective, the message to retain is that we need to avoid using pathogens with severe virulence under co-infections settings.

The second scenario of evolutionary suicide (see Figs. 9, 10) is more interesting from the eco-evolutionary point of view since it allows the persistence of all types of pathogens at the edge of extinction. The main benchmark of the considered scenario is the long-term stochastic persistence of species in the absence of a co-evolutionary attractor. Since at low virulence, the system shows a drift towards more severe strains, in the absence of a co-ESS, it would unavoidably reach the domain of extinction, where one or more types of the pathogen would go extinct. This is an example of the tragedy of commons phenomenon, where individual improvement of competing parties would eventually result in a collapse of the community^55^. However, if we admit an ecologically realistic situation, where dispersal from neighbour sites constantly re-introduces all pathogens species, the co-existence of parasites involved in the control will be still possible over evolutionary time. We need to assume that sites with host-parasite interactions are not synchronised in time and each type of pathogen persists at some site. In this case, temporal re-establishment of pathogens on a given site is guaranteed since the previous extinction via evolutionary suicide pushes the system to the parametric domain characterised by lower virulence allowing for the co-existence of all pathogens. We can assume a meta-community of local sites to be connected via dispersal. In the case of controlling the red palm weevil, this will be an assembly of plantations of palm trees in some fragmented landscape. For such a meta-community setting, stochastic persistence of all pathogen species becomes possible despite repeated evolutionary suicides at each local site. This scenario requires spatial heterogeneity in the system, where each local site is temporally dominated by a particular type of pathogen. Spatial synchronisation of eco-evolutionary dynamics would eventually lead to the persistence of only a single pathogen species in the considered meta-community. We should stress, however, that for the regime of stochastic co-existence of all types of pathogens at the edge of extinction, the increase of virulence under co-infection settings will be only marginal and will be limited by the boundary of the domain of co-existence of pathogens. As such, the presence of multiple pathogens in the environment, i.e. having a high bio-diversity of the system, would not have a pronounced positive effect on the control compared with the single infection scenario.

A key question is about the robustness of the outcomes obtained using the given specific model, specific trade-off functions and some simplifying assumptions, for example, the absence of direct competition between different parasite types inside the host body. To partially address the generality of our results, we verified the importance of a key assumption that only healthy individuals can reproduce. In particular, we also explored the scenario where the growth rate of the healthy host *S* given by $F(N_e)N_e$ (instead of *F*(*N*)*S*), where $N_e$ is the efficient number defined by Eq. (10). The underlying biological rationale is that the contribution to the growth rate by infected host individuals should be somehow weighted, taking into account the effects of debilitation of host individuals by co-infections, included in the expression for $N_e$. We found that considering the growth rate given by $F(N_e)N_e$ only slightly modifies our previous results. Moreover, considering the parameterisation of the growth rate of the host, given by $F(N)N_e$, we found only marginal changes in the graphs shown in the Results section. We also briefly investigated the scenario where the parameters in the virulence-transmission trade-off functions are distinctly different: we again observed qualitatively similar outcomes of biological control, indicating the robustness of our results.

The current theoretical study has its limitations, which can be addressed using some extended models. For example, unlike the study by Choisy and de Roode^25^, we did not consider the possibility of infected host recovery. Although our preliminary simulation showed that adding a small (but a constant) recovery term does not largely alter the main conclusions obtained with the model without recovery, the evolution of the host recovery rate may potentially affect the results, which is worth further investigation. Our model also ignores spatial heterogeneity, affecting epidemiological and evolutionary dynamics.^31,56,57^. An important further extension of this study would be modelling the effects of direct competition between different parasites inside the host, for example, interacting parasites can reduce their negative impact on the host by inhibiting each other^25,58,59^. Sometimes competition between parasites within the host can result in their competitive exclusion^59^. The current model assumes that all parasites are compatible and therefore able to infect the parasitized host, however, in several systems (as in viral hyperparasites infecting fungi), incompatibility builds a genetic barrier preventing infection of different varieties of the host^8,49^. Considering the effects of genetic incompatibility would be an important extension of this study. Furthermore, an interesting and ecologically relevant scenario of biological control using multiple enemies would be the one where some agents are parasites and others are predators^60^, which would combine trophic and parasitic interactions. It would be interesting to include in the model the possibility of co-evolution of the host, for example, by allowing for the evolution of the host resistance under co-infection settings (see the recent review by Buckingham and Ashby^61^ and references therein). Including relatedness of parasites (with the possibility of mutation between different parasite strains) and kin selection would be an important extension with several practical applications^46,62^. Finally, other questions, which were not covered in the current study, include alternative parasite transmission modes (i.e. considering the role of vertical transmission^8^ in the evolution of virulence) and the importance of considering the evolution process in fluctuating environment^63^.

## Supplementary Information

Supplementary Information.

## Data Availability

The datasets used and/or analysed during the current study available from the corresponding author on reasonable request.

## References

[1] Koppenhöfer A, Kaya H (1997). Additive and synergistic interaction between entomopathogenic nematodes andbacillus thuringiensisfor scarab grub control. Biol. Control.

[2] Wakil W, Yasin M, Shapiro-Ilan D (2017). Effects of single and combined applications of entomopathogenic fungi and nematodes against rhynchophorus ferrugineus (olivier). Sci. Rep..

[3] Hajek, A.E. & Eilenberg, J. *Natural enemies: An introduction to biological control* (Cambridge University Press, 2018).

[4] Perez-Alvarez R, Nault BA, Poveda K (2019). Effectiveness of augmentative biological control depends on landscape context. Sci. Rep..

[5] Ara ZG, Haque AR (2021). A comprehensive review on synthetic insecticides: Toxicity to pollinators, associated risk to food security, and management approaches. J. Biosyst. Eng..

[6] Kalha, C. *et al.* Entomopathogenic viruses and bacteria for insect-pest control. In *Integrated pest management*, 225–244 (Elsevier, 2014).

[7] Poveda J (2021). Trichoderma as biocontrol agent against pests: New uses for a mycoparasite. Biol. Control.

[8] Sandhu SK, Morozov AY, Holt RD, Barfield M (2021). Revisiting the role of hyperparasitism in the evolution of virulence. Am. Nat..

[9] Symondson W, Sunderland K, Greenstone M (2002). Can generalist predators be effective biocontrol agents?. Annu. Rev. Entomol..

[10] Louda SM, Pemberton R, Johnson M, Follett P (2003). Nontarget effects-the achilles’ heel of biological control? retrospective analyses to reduce risk associated with biocontrol introductions. Annu. Rev. Entomol..

[11] Tobin PC, Berec L, Liebhold AM (2011). Exploiting allee effects for managing biological invasions. Ecol. Lett..

[12] Van Lenteren J (2003). Environmental risk assessment of exotic natural enemies used in inundative biological control. Biocontrol.

[13] Dieckmann, U. Adaptive dynamics of pathogen-host interactions. *IIASA* (2002).

[14] Alizon S, Hurford A, Mideo N, Van Baalen M (2009). Virulence evolution and the trade-off hypothesis: History, current state of affairs and the future. J. Evol. Biol..

[15] Cressler CE, McLeod DV, Rozins C, Van Den Hoogen J, Day T (2016). The adaptive evolution of virulence: A review of theoretical predictions and empirical tests. Parasitology.

[16] Fenner, F., Ratcliffe, F. N. *et al.* Myxomatosis. *Myxomatosis.* (1965).

[17] Denoth M, Frid L, Myers JH (2002). Multiple agents in biological control: Improving the odds?. Biol. Control.

[18] Cardinale BJ, Harvey CT, Gross K, Ives AR (2003). Biodiversity and biocontrol: Emergent impacts of a multi-enemy assemblage on pest suppression and crop yield in an agroecosystem. Ecol. Lett..

[19] Alhadidi SN, Griffin JN, Fowler MS (2018). Natural enemy composition rather than richness determines pest suppression. Biocontrol.

[20] Dlamini BE, Malan AP, Addison P (2020). Combined effect of entomopathogenic fungi and steinernema yirgalemense against the banded fruit weevil, phlyctinus callosus (coleoptera: Curculionidae). Biocontrol Sci. Tech..

[21] Culshaw-Maurer M, Sih A, Rosenheim JA (2020). Bugs scaring bugs: Enemy-risk effects in biological control systems. Ecol. Lett..

[22] Abraham V, Shuaibi MA, Faleiro J, Abozuhairah R, Vidyasagar PS (1998). An integrated management approach for red palm weevil rhynchophorus ferrugineus oliv. a key pest of date palm in the middle east. J. Agric. Marine Sci. [JAMS].

[23] Kassem HS, Alotaibi BA, Ahmed A, Aldosri FO (2020). Sustainable management of the red palm weevil: The nexus between farmers’ adoption of integrated pest management and their knowledge of symptoms. Sustainability.

[24] Ansari M, Adhikari B, Ali F, Moens M (2008). Susceptibility of hoplia philanthus (coleoptera: Scarabaeidae) larvae and pupae to entomopathogenic nematodes (rhabditida: Steinernematidae, heterorhabditidae). Biol. Control.

[25] Choisy M, de Roode JC (2010). Mixed infections and the evolution of virulence: Effects of resource competition, parasite plasticity, and impaired host immunity. Am. Nat..

[26] Alizon S (2013). Co-infection and super-infection models in evolutionary epidemiology. Interface Focus.

[27] Bushman M, Antia R (2019). A general framework for modelling the impact of co-infections on pathogen evolution. J. R. Soc. Interface.

[28] Liu L, Ren X, Liu X (2020). A host-parasite system with multiple parasite strains and superinfection revisited: The global dynamics. Acta. Biotheor..

[29] van Baalen M, Sabelis MW (1995). The dynamics of multiple infection and the evolution of virulence. Am. Nat..

[30] Balmer O, Tanner M (2011). Prevalence and implications of multiple-strain infections. Lancet. Infect. Dis.

[31] Martcheva, M. *An introduction to mathematical epidemiology*, vol. 61 (Springer, 2015).

[32] Seabloom EW (2015). The community ecology of pathogens: Coinfection, coexistence and community composition. Ecol. Lett..

[33] Geritz SA, Metz JA, Kisdi É, Meszéna G (1997). Dynamics of adaptation and evolutionary branching. Phys. Rev. Lett..

[34] Brännström Å, Johansson J, Von Festenberg N (2013). The hitchhiker’s guide to adaptive dynamics. Games.

[35] Geritz SA, Mesze G, Metz JA (1998). Evolutionarily singular strategies and the adaptive growth and branching of the evolutionary tree. Evol. Ecol..

[36] Kisdi É, Stefan A, Geritz H (2010). Adaptive dynamics: A framework to model evolution in the ecological theatre. J. Math. Biol..

[37] Kisdi É, Priklopil T (2011). Evolutionary branching of a magic trait. J. Math. Biol..

[38] Alizon S, de Roode JC, Michalakis Y (2013). Multiple infections and the evolution of virulence. Ecol. Lett..

[39] May RM, Nowak MA (1995). Coinfection and the evolution of parasite virulence. Proc. Royal Soc. London Ser.: B Biol. Sci..

[40] Heesterbeek, H. The law of mass-action in epidemiology: A historical perspective. *Ecological Paradigms Lost: Routes of Theory Change* 81–104 (2005).

[41] Eshel I (1983). Evolutionary and continuous stability. J. Theor. Biol..

[42] Taylor PD (1989). Evolutionary stability in one-parameter models under weak selection. Theor. Popul. Biol..

[43] Christiansen FB (1991). On conditions for evolutionary stability for a continuously varying character. Am. Nat..

[44] Abrams PA, Matsuda H, Harada Y (1993). Evolutionarily unstable fitness maxima and stable fitness minima of continuous traits. Evol. Ecol..

[45] Okosun KO, Rachid O, Marcus N (2013). Optimal control strategies and cost-effectiveness analysis of a malaria model. Biosystems.

[46] Lion S (2013). Multiple infections, kin selection and the evolutionary epidemiology of parasite traits. J. Evol. Biol..

[47] Hussain A, Rizwan-ul Haq M, Al-Jabr AM, Al-Ayied HY (2013). Managing invasive populations of red palm weevil: A worldwide perspective. J. Food Agric. Environ..

[48] Gindin G, Levski S, Glazer I, Soroker V (2006). Evaluation of the entomopathogenic fungimetarhizium anisopliae andbeauveria bassiana against the red palm weevilrhynchophorus ferrugineus. Phytoparasitica.

[49] Milgroom MG, Cortesi P (2004). Biological control of chestnut blight with hypovirulence: A critical analysis. Annu. Rev. Phytopathol..

[50] Morozov AY, Robin C, Franc A (2007). A simple model for the dynamics of a host-parasite-hyperparasite interaction. J. Theor. Biol..

[51] Parvinen K (2005). Evolutionary suicide. Acta. Biotheor..

[52] Ferriere R, Legendre S (2013). Eco-evolutionary feedbacks, adaptive dynamics and evolutionary rescue theory. Philos. Trans. of the Royal Soc. B: Biol. Sci..

[53] Boldin B, Kisdi E (2016). Evolutionary suicide through a non-catastrophic bifurcation: Adaptive dynamics of pathogens with frequency-dependent transmission. J. Math. Biol..

[54] Henriques GJ, Osmond MM (2020). Cooperation can promote rescue or lead to evolutionary suicide during environmental change. Evolution.

[55] Rankin DJ, Bargum K, Kokko H (2007). The tragedy of the commons in evolutionary biology. Trends Ecol. Evol..

[56] Hesse E, Best A, Boots M, Hall A, Buckling A (2015). Spatial heterogeneity lowers rather than increases host-parasite specialization. J. Evol. Biol..

[57] Chabas H (2018). Evolutionary emergence of infectious diseases in heterogeneous host populations. PLoS Biol..

[58] Sallinen S, Susi H, Halliday F, Laine A-L (2022). Altered within-and between-host transmission under coinfection underpin parasite co-occurrence patterns in the wild. Evol. Ecol..

[59] Dutt A, Andrivon D, Le May C (2022). Multi-infections, competitive interactions, and pathogen coexistence. Plant. Pathol..

[60] Ong TWY, Vandermeer JH (2015). Coupling unstable agents in biological control. Nat. Commun..

[61] Buckingham LJ, Ashby B (2022). Coevolutionary theory of hosts and parasites. J. Evol. Biol..

[62] Buckling A, Brockhurst M (2008). Kin selection and the evolution of virulence. Heredity.

[63] Best A, Ashby B (2021). Evolutionarily stable strategies are well studied in periodically fluctuating populations. Proc. Natl. Acad. Sci..

