# Author Correction: Revisiting implementation of multiple natural enemies in pest management

**DOI:** 10.1038/s41598-022-26214-x

**Published:** 2022-12-14

**Authors:** Weam Alharbi, Simran K. Sandhu, Mounirah Areshi, Abeer Alotaibi, Mohammed Alfaidi, Ghada Al‑Qadhi, Andrew Yu Morozov

**Affiliations:** 1 Department of Mathematics, Faculty of Science, University of Tabuk, Tabuk, 71491 Saudi Arabia; 2 School of Computing and Mathematical Sciences, University of Leicester, Leicester, LE1 7RH UK; 3 Department of Biology, University College of Duba, University of Tabuk, Tabuk, 71491 Saudi Arabia; 4 Laboratory of Behaviour of Lower Vertebrates, Institute of Ecology and Evolution, Moscow, Russia 119071

Correction to: *Scientific Reports* 10.1038/s41598-022-18120-z, published online 02 September 2022

The original version of this Article contained an error in the Acknowledgments section.

“We gratefully acknowledge the support and the funding sources that are provided by the University of Tabuk (Saudi Arabia). We are thankful to Professor Sergei Petrovskii (University of Leicester, UK) for careful reading of the manuscript.”

now reads:

“We gratefully acknowledge the support and the funding sources that are provided by the University of Tabuk (Saudi Arabia) and the Vice-Presidency for Graduate Studies and Scientific Research for the project number (0148-1442-S). We are thankful to Professor Sergei Petrovskii (University of Leicester, UK) for careful reading of the manuscript. ”

The original Article has been corrected.

