# Supplementary material for: Revisiting implementation of multiple natural enemies in pest management
Source: Sci Rep. 2022 Sep 2;12:15023. doi: 10.1038/s41598-022-18120-z (PMC9440112; doi:10.1038/s41598-022-18120-z)
Supplement: Supplementary file 1 — Supplementary Information. [file 41598_2022_18120_MOESM1_ESM.pdf]

Supplementary Material: Revisiting implementation of multiple natural enemies in pest management

Weam Alharbi[1], Simran K. Sandhu[2], Mounirah Areshi[1], Abeer Alotaibi[1], Mohammed Alfaidi[3], Ghada Al-Qadhi[1], and Andrew Yu. Morozov[2,4*]

[1]Department of Mathematics, Faculty of science, University of Tabuk, Tabuk 71491, Saudi Arabia
[2]School of Computing and Mathematical Sciences, University of Leicester, LE1 7RH, UK
[3]Department of Biology, University College of Duba , University of Tabuk, Tabuk 71491, Saudi Arabia
[4]Laboratory of Behaviour of Lower Vertebrates, Institute of Ecology and Evolution, Moscow 119071, Russia
[*]Corresponding author; e-mail: am379@le.ac.uk

Contents

SM1 Single Infection Model **1**
SM1.1 Evolution with One Parasite Present . . . 2

SM2 Double Infection Model **2**
SM2.1 Evolution with a Single Parasite . . . 3
SM2.2 Co-Evolution of Two Co-infecting Parasites . . . 4

SM3 Triple Infection Model **5**

SM4 Quadruple Infection Model **6**

SM5 Cost-Effectiveness of Biological Control **7**

SM6 Calculating the prevalence of co-infections and the corresponding mortality rates. **9**

SM1 Single Infection Model

The model equations for S and I when we only have one type of parasite present are as follows

$$\frac{dS}{dt} = F(N)S - \mu S - S\beta_1^1 I_1, \tag{S1}$$

$$\frac{dI_1}{dt} = \beta_1^1 I_1 S - (\mu + \alpha_1) I_1. \tag{S2}$$

The stationary states of these are given as follows

$$S = \frac{\mu + \alpha_1}{\beta_1^1}. \tag{S3}$$

As we have $F(N) = r(1 - N/K)$,

$$I_1 = \frac{r - (r/K)S - \mu}{\beta_1^1 + r/K}. \tag{S4}$$

This results in

$$I_1 = \frac{r - (r/K)\frac{\mu+\alpha_1}{\beta_1^1} - \mu}{\beta_1^1 + (r/K)}, \tag{S5}$$

and

$$N = \frac{\alpha_1 + r}{\beta_1^1 + r/K}. \tag{S6}$$

SM1.1 Evolution with One Parasite Present

In this case our mutant equations are given as follows

$$\frac{dI_m}{dt} = \beta_m^m I_m S^* - (\mu + \alpha_m) I_m. \tag{S7}$$

This defines invasion fitness as

$$\lambda(\alpha_1, \alpha_m) = \beta_m^m S^* - (\mu + \alpha_m). \tag{S8}$$

It is straight-forward to derive that $S = \frac{\mu+\alpha_1}{\beta_2^2}$

$$\lambda(\alpha_1, \alpha_m) = (\mu + \alpha_1)\frac{\beta_m^m}{\beta_2^2} - (\mu + \alpha_m). \tag{S9}$$

We can also use this expression to locate the ESS.

$$\frac{\delta\lambda(\alpha*_m, \alpha*_m)}{\delta\alpha_m} = 0 = \frac{\mu + \alpha*_m}{\alpha*_m}\frac{K(K + \alpha*_m)}{(K + \alpha*_m)^2} - 1. \tag{S10}$$

This gives the ESS for a single strain as

$$\alpha_s^* = \sqrt{K\mu}. \tag{S11}$$

SM2 Double Infection Model

In the case of only two types of possible co-infections, the model equations for S, $I_j = I_{i_j}$ $(j = 1,2)$ and $D = I_{i_1,i_2}$ are as follows

$$\frac{dS}{dt} = F(N)S - \mu S - S\left(\beta_1^1 I_1 + \beta_2^2 I_2 + \left(\beta_{1,2}^1 + \beta_{1,2}^2\right)D\right), \tag{S12}$$

$$\begin{aligned}\frac{dI_1}{dt} &= \beta_1^1 I_1 S + \beta_{1,2}^1 DS - \beta_{1,2}^2 I_1 D - \beta_2^2 I_1 I_2 - (\mu + \alpha_1) I_1,\\ \frac{dI_2}{dt} &= \beta_2^2 I_2 S + \beta_{1,2}^2 DS - \beta_{1,2}^1 I_2 D - \beta_1^1 I_2 I_1 - (\mu + \alpha_2) I_2,\end{aligned} \tag{S13}$$

$$\frac{dD}{dt} = \beta_{1,2}^1 I_1 D + \beta_{1,2}^2 I_2 D + \left(\beta_1^1 + \beta_2^2\right) I_1 I_2 - (\mu + \alpha_{1,2}) D. \tag{S14}$$

A detailed flowchart of the model is provided in Figure S1.

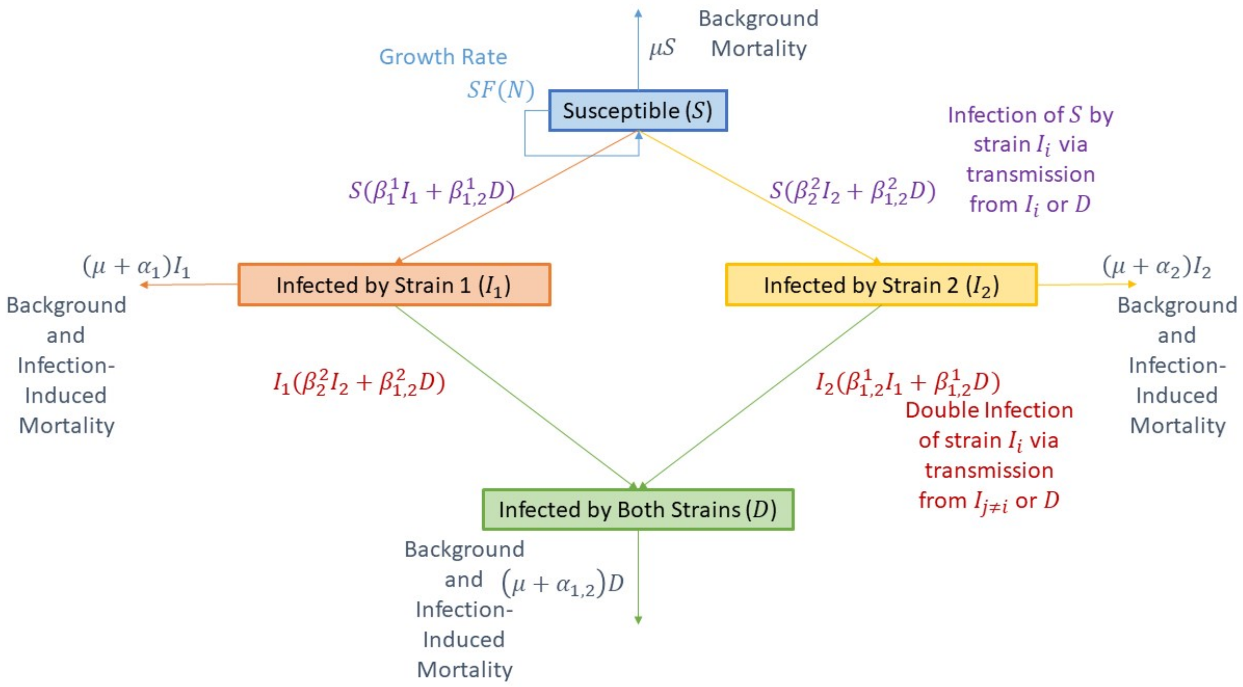

Figure S1. Flowchart displaying all interactions within the generic model that accounts for the existence of multiple strains of parasite, given by equations (S12)-(S14). The system consists of three components; healthy hosts (S), hosts infected by a parasite of strain i only (I_i, $i = 1,2$) and hosts infected by both strains of parasite (D).

SM2.1 Evolution with a Single Parasite

The model flowchart of this is shown in Figure S2, in terms of the adaptive dynamics framework, we consider S, I_1, I_2 and D to be at the stationary states and therefore we just model the rare mutant strains of I_2, denoted as I_m and D_{1m}. As the mutant strains are so rare we consider $I_m D_{1m} \approx 0$ simplifying the invasion model to the following

$$\frac{dI_m}{dt} = \beta_m^m I_m S^* + \beta_{1,2_m}^m D_{1m} S^* - \beta_{1,2}^1 I_m D^* - \beta_1^1 I_m I_1^* - (\mu + \alpha_{2_m}) I_m, \tag{S15}$$

$$\frac{dD_{1m}}{dt} = \beta_{1,2_m}^m I_1^* D_{1m} + \beta_{1,2}^1 D^* I_m + \left(\beta_1^1 + \beta_m^m\right) I_1^* I_m - (\mu + \alpha_{1,2_m}) D_{1m}. \tag{S16}$$

In matrix form this is given as follows

$$\begin{bmatrix} \frac{dI_m}{dt} \\ \frac{dD_{1m}}{dt} \end{bmatrix} = \begin{bmatrix} \beta_m^m S^* - \beta_{1,2}^1 D^* - \beta_1^1 I_1^* - (\mu + \alpha_{2_m}) & \beta_{1,m}^m S^* \\ \beta_{1,2}^1 D^* + \left(\beta_1^1 + \beta_m^m\right) I_1^* & \beta_{m,1}^m I_1^* - (\mu + \alpha_{1,2_m}) \end{bmatrix} \begin{bmatrix} I_m \\ D_{1m} \end{bmatrix} , \tag{S17}$$

which gives the following characteristic equation

$$\left(\beta_m^m S^* - \beta_{1,2}^1 D^* - \beta_1^1 I_1^* - (\mu + \alpha_m) - \lambda\right) \left(\beta_{1,m}^1 I_1^* - (\mu + \alpha_{1,2_m}) - \lambda\right) - \beta_{1,m}^m S^* (\beta_{1,2}^1 D^* + \left(\beta_1^1 + \beta_m^m\right) I_1^*) = 0. \tag{S18}$$

From which we can obtain $\lambda_{1,2}$, we take the invasion fitness to be $\lambda(\alpha_2, \alpha_m) = \max(\lambda_{1,2})$.

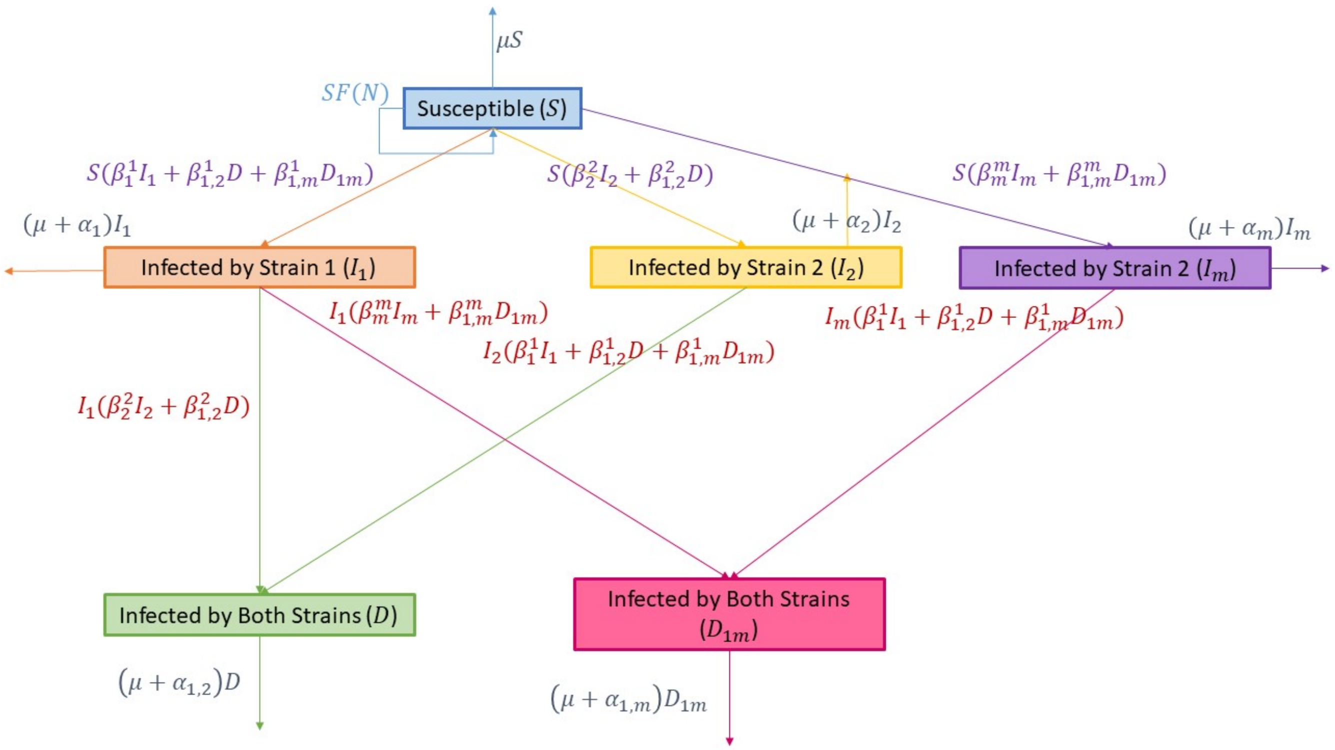

Figure S2. Flowchart displaying all interactions within the generic model that accounts for the existence of multiple strains of parasite, given by equations (S12)-(S14). The system consists of three components; healthy hosts (S), hosts infected by a parasite of strain i only (I_i, $i = 1, 2$) and hosts infected by both strain of parasite (D).

SM2.2 Co-Evolution of Two Co-infecting Parasites

We also consider the case when we allow both strains of the parasite to evolve, i.e. co-evolution. The flowchart for this scenario is shown in Figure S3.

In terms of the adaptive dynamics framework, we consider S, I_1, I_2 and D to be at the stationary states and therefore we just model the mutant strains;

$$\frac{dI_{m_1}}{dt} = \beta^{1m}_{1_m} I_{m_1} S^* + \beta^{1m}_{1_m,2_m} D_{m_1 m_2} S^* + \beta^{1m}_{1_m,2} D_{m_1 2} S^* - I_{m_1}(\beta^2_2 I^*_2 + \beta^2_{1,2} D^*_{12}) - (\mu + \alpha_{1_m}) I_{m_1}, \tag{S19}$$

$$\frac{dI_{m_2}}{dt} = \beta^{2m}_{2_m} I_{m_2} S^* + \beta^{2m}_{1_m,2_m} D_{m_1 m_2} S^* + \beta^{2m}_{1,2_m} D_{1 m_2} S^* - I_{m_2}(\beta^1_1 I^*_1 + \beta^1_{12} D^*_{12}) - (\mu + \alpha_{2_m}) I_{m_2}, \tag{S20}$$

$$\frac{dD_{1m_2}}{dt} = I^*_1(\beta^{2m}_{2_m} I_{m2} + \beta^{2m}_{1,2_m} D_{1m_2} + \beta^{2m}_{1_m,2_m} D_{m_1 m_2}) + I_{m_2}(\beta^1_1 I^*_1 + \beta^1_{1,2} D^*_{12}) - (\mu + \alpha_{1,2_m}) D_{1m_2}, \tag{S21}$$

$$\frac{dD_{m_1 2}}{dt} = I^*_2(\beta^{1m}_{1_m} I_{m_1} + \beta^{1m}_{1_m,2} D_{m_1 2} + \beta^{1m}_{1_m,2_m} D_{m_1 m_2}) + I_{m_1}(\beta^2_2 I^*_2 + \beta^2_{1,2} D^*_{12}) - (\mu + \alpha_{1_m,2}) D_{m_1 2}, \tag{S22}$$

$$\frac{dD_{m_1 m_2}}{dt} = -(\mu + \alpha_{1_m,2_m}) D_{m_1 m_2}. \tag{S23}$$

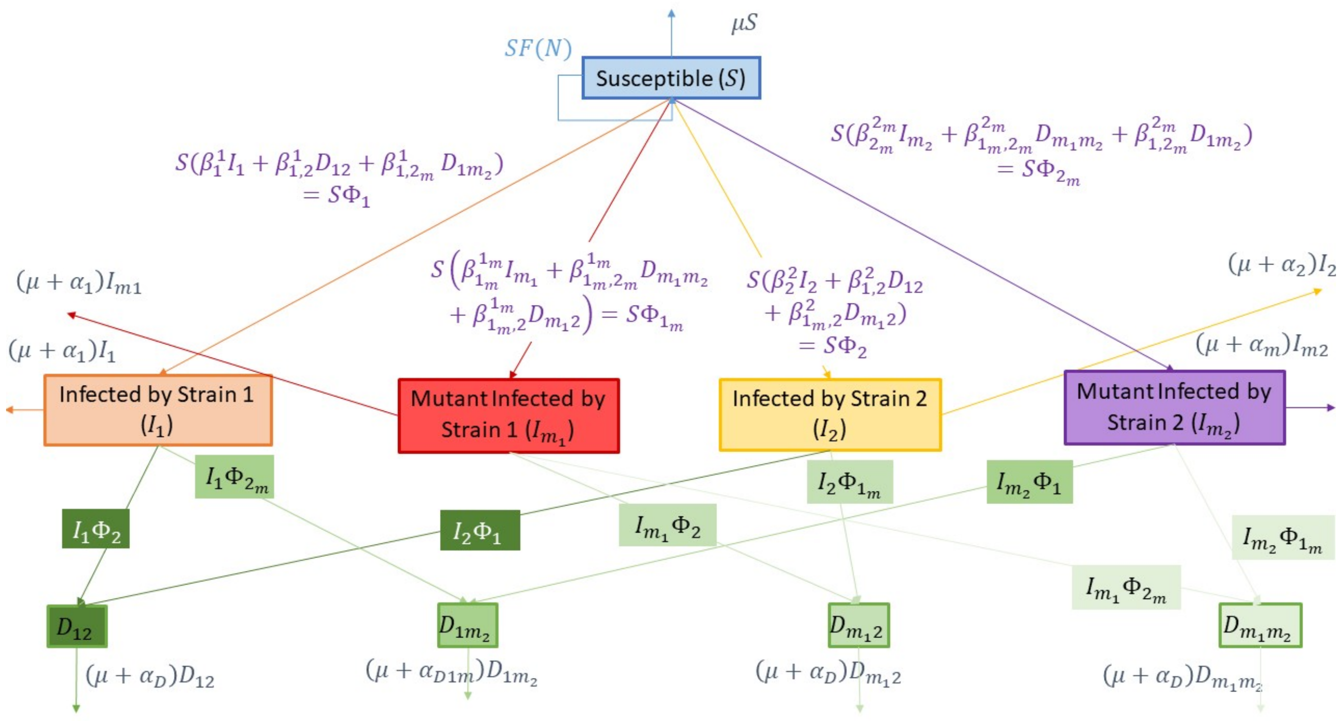

Figure S3. Flowchart displaying all interactions within the generic model that accounts for the existence of multiple strains of parasite with the invasion of a mutant of both strains, given by equations (S19)-(S23). The system consists of three components; healthy hosts (S), hosts infected by a parasite of strain i only (I_i, $i = 1, 2, m_1, m_2$) and hosts infected by two strains of parasite ($D_{i,j\neq i}$).

Again, note that due to how rare the mutants are $I_{m_1}D_{m_1} = I_{m_1}D_{m_2} = I_{m_2}D_{m_2} = I_{m_2}D_{m_1} = 0$. From this model we use $\lambda_{m1} = \max(\lambda_1, \lambda_4)$ and $\lambda_{m2} = \max(\lambda_2, \lambda_3)$ as the invasion fitness for I_{m_1} and I_{m_2}, respectively. As $\frac{dD_{m_1m_2}}{dt} < 0$ we can neglect it from out analysis.

SM3 Triple Infection Model

Now consider the case when we have three possible types of parasite; I_{i_j} (denoted here by I_j, $j = 1, 2, 3$) which can pairwise doubly infect hosts; I_{i_j,i_k} (denoted here by D_{12}, D_{13} and D_{23}), furthermore, the host can be triply infected by all three types I_{i_1,i_2,i_3} (simply denoted by T). The model for this scenario can be described by the following set of equations

$$\frac{dS}{dt} = F(N)S - \mu S - S(\Phi_1 + \Phi_2 + \Phi_3), \tag{S24}$$

$$\begin{aligned}
\frac{dI_1}{dt} &= \Phi_1 - I_1\left(\Phi_2 + \Phi_3\right) - (\mu + \alpha_1)I_1, \\
\frac{dI_2}{dt} &= \Phi_2 - I_2\left(\Phi_1 + \Phi_3\right) - (\mu + \alpha_2)I_2, \\
\frac{dI_3}{dt} &= \Phi_3 - I_3\left(\Phi_1 + \Phi_2\right) - (\mu + \alpha_3)I_3,
\end{aligned} \tag{S25}$$

$$\frac{dI_{1,2}}{dt} = \frac{dD_{12}}{dt} = I_1\Phi_2 + I_2\Phi_1 - D_{12}\left(\Phi_3\right) - (\mu + \alpha_{1,2})\,D_{12},$$
$$\frac{dI_{1,3}}{dt} = \frac{dD_{13}}{dt} = I_1\Phi_3 + I_3\Phi_1 - D_{13}\left(\Phi_2\right) - (\mu + \alpha_{1,3})\,D_{13}, \quad \text{(S26)}$$
$$\frac{dI_{2,3}}{dt} = \frac{dD_{23}}{dt} = I_2\Phi_3 + I_3\Phi_2 - D_{23}\left(\Phi_1\right) - (\mu + \alpha_{2,3})\,D_{23},$$

$$\frac{dI_{1,2,3}}{dt} = \frac{dT}{dt} = D_{12}\Phi_3 + D_{13}\Phi_2 + D_{23}\Phi_1 - (\mu + \alpha_{1,2,3})\,T, \quad \text{(S27)}$$

where

$$\Phi_1 = I_1\beta_1^1 + \beta_{1,2}^1 D_{12} + \beta_{1,3}^1 D_{13} + \beta_{1,2,3}^1 T,$$
$$\Phi_2 = I_2\beta_2^2 + \beta_{1,2}^2 D_{12} + \beta_{2,3}^2 D_{23} + \beta_{1,2,3}^2 T, \quad \text{(S28)}$$
$$\Phi_3 = I_3\beta_3^3 + \beta_{1,3}^3 D_{13} + \beta_{2,3}^3 D_{13} + \beta_{1,2,3}^3 T,$$

SM4 Quadruple Infection Model

Finally, consider the case when we have four possible types of parasite; singly infected hosts I_{i_j} (denoted here by $I_j,\ j = 1,2,3c,4$) which can pairwise doubly infect hosts; I_{i_j,i_k} (denoted here by D_{12}, D_{13}, D_{14}, D_{23}, D_{24} and D_{34}), the host can be triply infected by all three types I_{i_1,i_2,i_3} (denoted by T_{123}, T_{124}, T_{134} and T_{234}) and quadruply infected hosts I_{i_1,i_2,i_3,i_4} (simply denoted by Q). The model for this scenario can be described by the following set of equations

$$\frac{dS}{dt} = F(N)S - \mu S - S\left(\Phi_1 + \Phi_2 + \Phi_3 + \Phi_4\right), \quad \text{(S29)}$$

$$\frac{dI_1}{dt} = \Phi_1 - I_1\left(\Phi_2 + \Phi_3 + \Phi_4\right) - (\mu + \alpha_1)\,I_1,$$
$$\frac{dI_2}{dt} = \Phi_2 - I_2\left(\Phi_1 + \Phi_3 + \Phi_4\right) - (\mu + \alpha_2)\,I_2,$$
$$\frac{dI_3}{dt} = \Phi_3 - I_3\left(\Phi_1 + \Phi_2 + \Phi_4\right) - (\mu + \alpha_3)\,I_3, \quad \text{(S30)}$$
$$\frac{dI_4}{dt} = \Phi_4 - I_4\left(\Phi_1 + \Phi_2 + \Phi_3\right) - (\mu + \alpha_4)\,I_4,$$

$$\frac{dD_{12}}{dt} = I_1\Phi_2 + I_2\Phi_1 - D_{12}\left(\Phi_3 + \Phi_4\right) - (\mu + \alpha_{1,2})\,D_{12},$$
$$\frac{dD_{13}}{dt} = I_1\Phi_3 + I_3\Phi_1 - D_{13}\left(\Phi_2 + \Phi_4\right) - (\mu + \alpha_{1,3})\,D_{13},$$
$$\frac{dD_{14}}{dt} = I_1\Phi_4 + I_4\Phi_1 - D_{14}\left(\Phi_2 + \Phi_3\right) - (\mu + \alpha_{1,4})\,D_{14},$$
$$\frac{dD_{23}}{dt} = I_2\Phi_3 + I_3\Phi_2 - D_{23}\left(\Phi_1 + \Phi_4\right) - (\mu + \alpha_{2,3})\,D_{23}, \tag{S31}$$
$$\frac{dD_{24}}{dt} = I_2\Phi_4 + I_4\Phi_2 - D_{24}\left(\Phi_1 + \Phi_3\right) - (\mu + \alpha_{2,4})\,D_{24},$$
$$\frac{dD_{34}}{dt} = I_3\Phi_4 + I_4\Phi_3 - D_{34}\left(\Phi_1 + \Phi_2\right) - (\mu + \alpha_{3,4})\,D_{34},$$

$$\frac{dT_{123}}{dt} = D_{12}\Phi_3 + D_{13}\Phi_2 + D_{23}\Phi_1 - T_{123}\Phi_4 - (\mu + \alpha_{1,2,3})\,T_{123},$$
$$\frac{dT_{134}}{dt} = D_{13}\Phi_4 + D_{14}\Phi_3 + D_{34}\Phi_1 - T_{134}\Phi_2 - (\mu + \alpha_{1,3,4})\,T_{134}, \tag{S32}$$
$$\frac{dT_{124}}{dt} = D_{12}\Phi_4 + D_{14}\Phi_2 + D_{24}\Phi_1 - T_{124}\Phi_3 - (\mu + \alpha_{1,2,4})\,T_{124},$$
$$\frac{dT_{234}}{dt} = D_{23}\Phi_4 + D_{24}\Phi_3 + D_{34}\Phi_2 - T_{234}\Phi_1 - (\mu + \alpha_{2,3,4})\,T_{234},$$

$$\frac{dQ}{dt} = T_{123}\Phi_4 + T_{124}\Phi_3 + T_{134}\Phi_2 + T_{234}\Phi_1 - (\mu + \alpha_{1,2,3,4})\,Q, \tag{S33}$$

where

$$\Phi_1 = I_1\beta_1^1 + \beta_{1,2}^1 D_{12} + \beta_{1,3}^1 D_{13} + \beta_{1,4}^1 D_{14} + \beta_{1,2,3}^1 T_{123} + \beta_{1,2,4}^1 T_{124} + \beta_{1,3,4}^1 T_{134} + \beta_{1,2,3,4}^1 Q,$$
$$\Phi_2 = I_2\beta_2^2 + \beta_{1,2}^2 D_{12} + \beta_{2,3}^2 D_{23} + \beta_{2,4}^2 D_{24} + \beta_{1,2,3}^2 T_{123} + \beta_{1,2,4}^2 T_{124} + \beta_{2,3,4}^2 T_{234} + \beta_{1,2,3,4}^2 Q, \tag{S34}$$
$$\Phi_3 = I_3\beta_3^3 + \beta_{1,3}^3 D_{13} + \beta_{2,3}^3 D_{23} + \beta_{3,4}^3 D_{34} + \beta_{1,2,3}^3 T_{123} + \beta_{1,3,4}^3 T_{134} + \beta_{2,3,4}^3 T_{234} + \beta_{1,2,3,4}^3 Q,$$
$$\Phi_4 = I_4\beta_4^4 + \beta_{1,4}^4 D_{14} + \beta_{2,4}^4 D_{24} + \beta_{3,4}^4 D_{34} + \beta_{1,2,4}^4 T_{124} + \beta_{1,3,4}^4 T_{134} + \beta_{2,3,4}^4 T_{234} + \beta_{1,2,3,4}^4 Q,$$

SM5 Cost-Effectiveness of Biological Control

In this study, we consider two measures of cost-effectiveness of biological control by co-infecting parasites: the average cost-effectiveness ratio (ACER) and the incremental cost-effectiveness ratio (ICER), for details of the formal definitions, see the main text. Figures 3 (C,D) of the main text show the values of ACER. In figure S4, we show the graphs of the ICER corresponding to the parameters in Figure 3 of the main text. The negative value for the ICER observed for $n = 4$ in panel (A) signifies a decrease in the effectiveness with an increase in the cost while transferring from the triple to the quadruple co-infection biological control.

Finally, we analyse the cost-effectiveness of biological control according to the scenario, where the efficient population size corresponds to the total number (density) of the susceptible host, i.e. $N_e = S$. In this case, the effectiveness is measured as $(S(0) - S(n))/S(0)$, and the increment in effectiveness - when adding an extra type of natural enemy in the system to the existing $n-1$ types - is defined as $(S(n-1) - S(n))/S(0)$. The corresponding graphs for both the ICER and the ACER are shown in Figure S5.

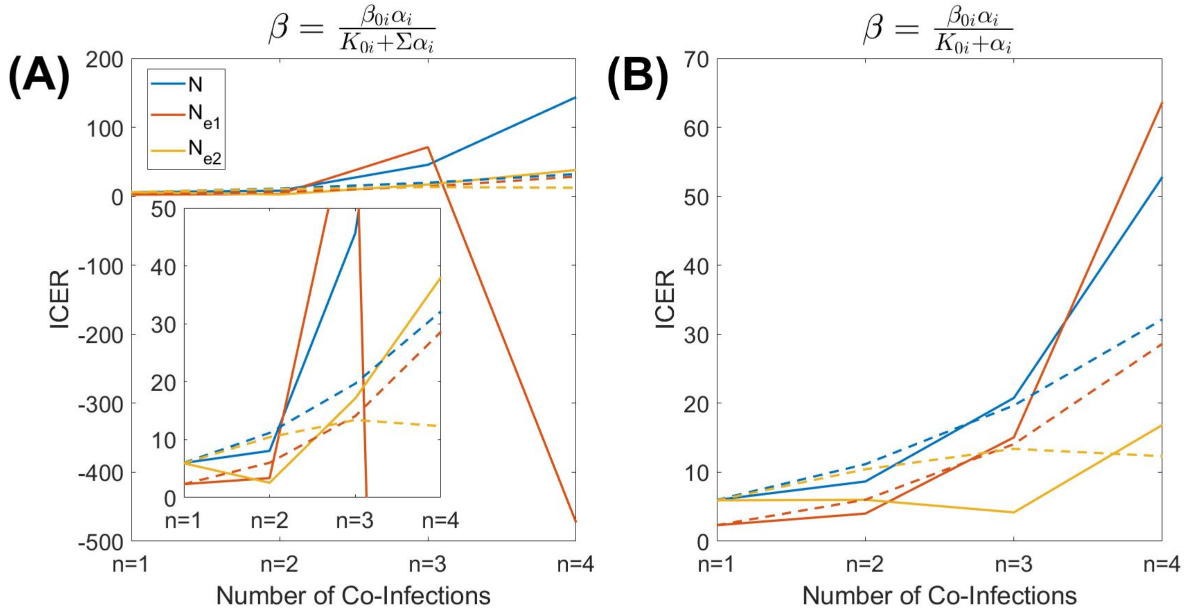

Figure S4. Dependence of the ICER on the possible number of co-infections n for two cases of transmission $\beta^{i_j}_{i_1,i_2,\ldots,i_k}$. The blue curves display the ICER when effectiveness is evaluated using the total species density N at the co-ESS virulence (mathematically, the increment in effectiveness, when adding an extra type of natural enemy to the existing $n-1$ types is given by $(N(n-1)-N(n))/N(0)$). The orange and yellow curves display the ICER based on the efficient population size of the pest N_e at the ESS virulence (mathematically, the increment in effectiveness, when adding an extra type of natural enemy to the existing $n-1$ types, is given by $(N_e(n-1)-N_e(n))/N_e(0)$), when weighting functions w_1 and w_2 are used. The dashed curves correspond to the short-time scale scenario, with the virulence given by the ESS for a single infection ($n=1$). Within the weighting functions w_1 and w_2, the parameters are as follows; $\alpha_0 = 0.4$, $a = 1$, $b = 20$ and $c = 5$, all other model parameters are as defined in Table 1.

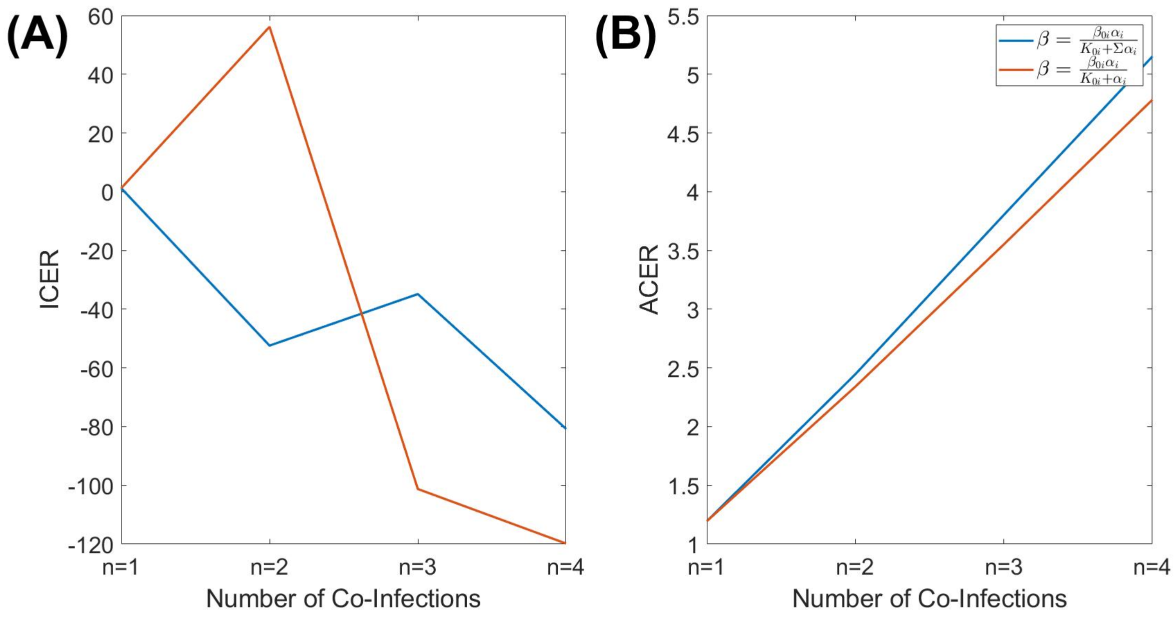

Figure S5. Dependence of (A) the ICER and (B) the ACER on the possible number of co-infections n for two scenarios of transmission $\beta^{i_j}_{i_1,i_2,...,i_k}$ in the case when the effectiveness is evaluated based on the total susceptible host density S at the co-ESS virulence. The dashed curves correspond to the short-time scale scenario, with the virulence given by the ESS for a single infection ($n = 1$). Within the weighting functions w_1 and w_2, the parameters are as follows; $\alpha_0 = 0.4$, $a = 1$, $b = 20$ and $c = 5$, all other model parameters are as defined in Table 1.

SM6 Calculating the prevalence of co-infections and the corresponding mortality rates.

In this section we evaluate the values co-infection prevalence $p(I_{i_1,i_2,...,i_k})$ $(i_j = 1,...,n)$ for the system at equilibrium. To find the equilibrium values of host density, we use the model simulation in the main text. For each maximal number n of possible co-infections, the prevalence of a host having a particular number of co-infections is determined by the following proportion

$$p(I_{i_1,i_2,...,i_k}) = \frac{I_{i_1,i_2,...,i_k}}{N}, \tag{S35}$$

where N is the total number of hosts, in the above expression below we understand all densities as stationary ones.

Table 1 presents the co-infection prevalence values corresponding to the scenarios shown in Figure 6 of the main text, where S, I, D, T, Q denote, respectively, the total population size (the total density) of the host infected by $k = 0$, $k = 1$, $k = 2$, $k = 3$, and $k = 4$ different types of parasites with $k \leq n$, n being the total maximal number of co-infections (e.g. $I = I_1 + I_2 + ...I_n$).

	$\beta = \frac{\beta_{0i}\alpha_i}{K_{0i}+\Sigma\alpha_i}$					$\beta = \frac{\beta_{0i}\alpha_i}{K_{0i}+\alpha_i}$				
	n=0	n=1	n=2	n=3	n=4	n=0	n=1	n=2	n=3	n=4
p(S)	1	0.1961	0.2573	0.3073	0.3286	1	0.1961	0.2028	0.2322	0.2518
p(I)	-	0.8039	0.3432	0.322	0.312	-	0.8039	0.2964	0.272	0.2668
p(D)	-	-	0.3995	0.2547	0.2243	-	-	0.5008	0.281	0.2416
p(T)	-	-	-	0.116	0.1086	-	-	-	0.2148	0.1692
p(Q)	-	-	-	-	0.0265	-	-	-	-	0.0706

Table 1. Prevalence (proportion) of host containing various numbers of co-infections (n the maximal number of co-infections), for the two different cases of transmission rates, calculated based upon the stationary densities displayed in Figure 6 of the main text.

In this section, we also calculate the probability that a host will die after having acquired exactly n infections (for generality, we consider $n = 0$, i.e. no infections, as well). For the system at equilibrium, mentioned probabilities can be calculated based on the following expression

$$p_m(I_{i_1,i_2,\ldots,i_k}) = \frac{(\mu + \alpha_{i_1,i_2,\ldots,i_k})I_{i_1,i_2,\ldots,i_k}}{\mu S + \sum(\mu + \alpha_{i_1,i_2,\ldots,i_k})I_{i_1,i_2,\ldots,i_k}} = \frac{(\mu + \alpha_{i_1,i_2,\ldots,i_k})I_{i_1,i_2,\ldots,i_k}}{F(N)S}, \tag{S36}$$

where μ is the background mortality, $\alpha_{i_{k_1},i_{k_2},\ldots,i_{k_n}}$ is the virulence due to infection by parasites $i_{k_1}, i_{k_2}, \ldots, i_{k_n}$. The rationale behind the above expression is that for the system at equilibrium, the reproduction rate (inflow) of the host should be compensated by the losses due to mortality. On the other hand, the number of hosts dying after acquiring particular infections $i_{k_1}, i_{k_2}, \ldots, i_{k_n}$ is proportional to the magnitude of the corresponding mortality rate, i.e. it is proportional to $(\mu + \alpha_{i_1,i_2,\ldots,i_k})I_{i_1,i_2,\ldots,i_k}$.

Importantly, the above expression defines the probability that a host will be infected by the maximal number of co-infections n, i.e. dies after acquiring all possible types of co-infections:

$$p_m(I_{1,2,3,\ldots,n}) = \frac{(\mu + \alpha_{1,2,3,\ldots,n})I_{1,2,3,\ldots,n}}{F(N)S}.$$

Using the above equations for p_m, we calculated the probabilities to die for host individuals as a result of acquiring exactly k infections, $k \leq n$. The results are presented in Table 2 which corresponds to modelling graphs shown in Figure 6 of the main text.

	$\beta = \frac{\beta_{0i}\alpha_i}{K_{0i}+\Sigma\alpha_i}$					$\beta = \frac{\beta_{0i}\alpha_i}{K_{0i}+\alpha_i}$				
	n=0	n=1	n=2	n=3	n=4	n=0	n=1	n=2	n=3	n=4
$p_m(S)$	1	0.1087	0.0655	0.0612	0.0599	1	0.1087	0.0673	0.0581	0.0551
$p_m(I)$	-	0.89131	0.31135	0.2828	0.2708	-	0.8913	0.251	0.206	0.1937
$p_m(D)$	-	-	0.6232	0.3966	0.3485	-	-	0.6817	0.3554	0.298
$p_m(T)$	-	-	-	0.2594	0.2431	-	-	-	0.3805	0.2945
$p_m(Q)$	-	-	-	-	0.0777	-	-	-	-	0.1587

Table 2. Probabilities of host depth after having acquired k infections, $k \leq n$ ($k = 0,1,2,3,4$ correspond to S, I, D, T, Q, respectively). Two different cases of transmission rate are considered, the stationary densities of species are the same as displayed in Figure 6 of the main text.

A comparison of Table 1 and Table 2 shows that the probability to die after k co-infections p_m can be substantially different from the corresponding values of co-infection prevalence p for the same number of co-infections. This is especially true for the maximal number of co-infections $k = n$. The mentioned discrepancy can be explained by an increasing mortality rate of hosts with a large number of co-infections.
